# Sampling re-design increases power to detect change in the Great Barrier Reef’s inshore water quality

**DOI:** 10.1371/journal.pone.0271930

**Published:** 2022-07-28

**Authors:** Luke R. Lloyd-Jones, Petra M. Kuhnert, Emma Lawrence, Stephen E. Lewis, Jane Waterhouse, Renee K. Gruber, Frederieke J. Kroon

**Affiliations:** 1 Data61, Commonwealth Scientific and Industrial Research Organisation, Brisbane, Queensland, Australia; 2 Centre for Tropical Water & Aquatic Ecosystem Research, James Cook University, Townsville, Queensland, Australia; 3 Australian Institute of Marine Science, Townsville, Queensland, Australia; Đại Học Duy Tân: Dai Hoc Duy Tan, VIET NAM

## Abstract

Monitoring programs are fundamental to understanding the state and trend of aquatic ecosystems. Sampling designs are a crucial component of monitoring programs and ensure that measurements evaluate progress toward clearly stated management objectives, which provides a mechanism for adaptive management. Here, we use a well-established marine monitoring program for inshore water quality in the Great Barrier Reef (GBR), Australia to investigate whether a sampling re-design has increased the program’s capacity to meet its primary objectives. Specifically, we use bootstrap resampling to assess the change in statistical power to detect temporal water quality trends in a 15-year inshore marine water quality data set that includes data from both before and after the sampling re-design. We perform a comprehensive power analysis for six water quality analytes at four separate study areas in the GBR Marine Park and find that the sampling re-design (i) increased power to detect trends in 23 of the 24 analyte-study area combinations, and (ii) resulted in an average increase in power of 34% to detect increasing or decreasing trends in water quality analytes. This increase in power is attributed more to the addition of sampling locations than increasing the sampling rate. Therefore, the sampling re-design has substantially increased the capacity of the program to detect temporal trends in inshore marine water quality. Further improvements in sampling design need to focus on the program’s capability to reliably detect trends within realistic timeframes where inshore improvements to water quality can be expected to occur.

## Introduction

Monitoring programs are fundamental to understanding the state and trend of ecosystems and to assessing the abundance, distribution and occurrence of biota or concentrations of chemical and biological parameters. A crucial component of scientifically robust monitoring programs is that the sampling design ensures measurements evaluate progress toward clearly stated management objectives [1–4]. Recent environmental studies that assessed the influence of sampling design on detecting change include Wagner et al. [5] who looked at the statistical power to detect temporal trends in riverine contaminants in the Chesapeake Bay Watershed. Further, O’Hare et al. [6] simulated the effect of network re-design on the statistical power to detect long-term trends across a national monitoring network of three key ecological indicators. Data collected within a rigorously designed monitoring program provide sufficient statistical power to assess change in indicators aligned with monitoring objectives, providing a mechanism for management intervention or adjustments [1, 7, 8]. Low power indicates a high probability of concluding that no environmental impact has occurred when in reality it has, resulting in substantial short- and long-term costs due to necessary action not being taken [1]. Optimal sampling designs balance the power and precision of primary parameters against budget, resource and practical constraints in an integrative and holistic manner [6, 9, 10]. Recently, O’Hare et al. [6] investigated the optimisation of statistical power to detect long-term trends when re-designing existing large-scale environmental monitoring networks. However, we are unaware of any studies that have used long-term monitoring data from both before and after a sampling re-design to evaluate whether the intervention has improved power to meet the program’s objectives.

The Great Barrier Reef (GBR) Marine Monitoring Program (MMP) is a well-established monitoring program with sufficient data to investigate the effect of sampling re-design on the statistical power to detect trends in marine water quality. The MMP monitors the health of inshore ecosystems in the GBR Marine Park, including coral reefs (Thompson et al., 2014), seagrass meadows [11], and water quality [12] to assess progress against the objectives of the Reef 2050 Long Term Sustainability Plan [13] and the Reef 2050 Water Quality Improvement Plan [14]. The MMP Inshore Water Quality component (MMP WQ), the focus of this study, was first established in 2005 [15] and builds on monitoring activities conducted in the GBR Marine Park since the early 1990s [16, 17]. The objectives of the MMP WQ have evolved since 2005; however, the aim of assessing temporal and spatial trends in inshore marine water quality has remained the program’s primary objective [12, 15]. The current overarching objective of the MMP WQ is to assess temporal and spatial trends in inshore marine water quality and to link pollutant concentrations to end-of-catchment loads [18]. From 2005 to 2014, the MMP WQ consisted of two main components: (i) since 2005, monitoring of ambient water quality using grab samples and data-logging instruments [19]; and (ii) since 2007, monitoring of flood plumes (resulting from river flood events) in the coastal ocean using water quality grab samples and remote sensing [20].

Following a critical review of the statistical design for the overall MMP, Kuhnert et al. [21] provided a set of considerations to the Great Barrier Reef Marine Park Authority (GBRMPA) that included a more comprehensive sampling design to improve the delivery of the MMP WQ monitoring objectives. Specifically, for the ambient water quality sampling component of the MMP WQ, which is the focus of this study, Kuhnert et al.’s [21] advice concentrated on the sampling design’s spatial representativeness and the capacity to link water quality data with other components of the program. In February 2015, GBRMPA, in collaboration with research partners, implemented the current MMP program, which included a re-design that added additional sampling locations and increased within-region sampling frequency [12].

Here, we aim to close an iteration of the adaptive monitoring cycle [4] by evaluating whether the MMP WQ sampling re-design has increased the statistical power to meet the program’s objective of assessing trends in inshore ambient water quality. Specifically, we use a bootstrap resampling algorithm [22, 23] to perform a comprehensive evaluation of power to detect trends for six water quality analytes between the 2005–2014 and the 2015–2019 sampling designs at four separate study areas. Furthermore, we statistically examine the effect of increasing the sampling frequency versus adding additional sampling locations on the power to detect a change in water quality trends.

## Materials and methods

### Great Barrier Reef

The GBR is the most extensive coral reef system in the world, extending for 2,300 km along Australia’s north-eastern coast [24–26]. The GBR region has been protected as the 344,400 km^2^ GBR Marine Park since 1975 and inscribed as the 348,000 km^2^ GBR World Heritage Area by UNESCO in 1981. The GBR World Heritage Area contains approximately 20,000 km^2^ of coral reefs, 43,000 km^2^ of seagrass meadows, 25,600 km^2^ of shoals, and extensive mangrove forests [26]. Despite a high level of protection, the condition of GBR ecosystems has deteriorated over the past decades (Great Barrier Reef Marine Park Authority, 2019) due to the combined impacts of mass coral bleaching events [27, 28], Crown-of-Thorns starfish outbreaks [29], and severe tropical cyclones [30], as well as poor inshore water quality [31–33].

### Study areas

**Fig 1:**
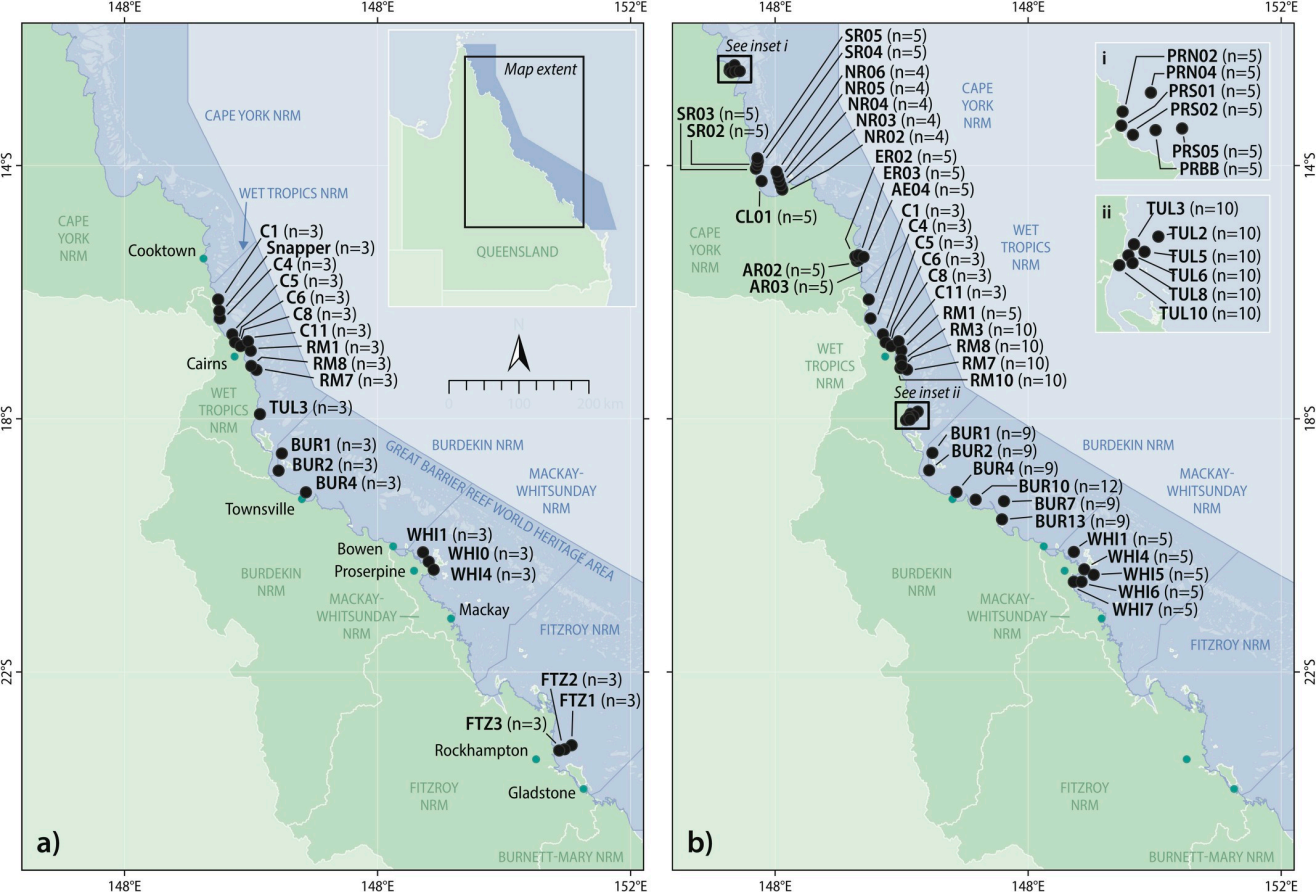
Survey locations of the Marine Monitoring Program for Inshore Water Quality from (a) 2005–2014 and (b) 2015–2019. The values of *n* refer to the number of samples taken per year across the ten-year and five-year sampling periods respectively. The dark blue area represents the Great Barrier Reef World Heritage Area; the dark blue lines within the GBR Marine Park represent the six marine Natural Resource Management (NRM) regions. Locations used in the current study are detailed in Table 1.

The current MMP WQ monitors the inshore waters of the GBR Marine Park across five of the six Natural Resource Management (NRM) regions: Cape York, Wet Tropics, Burdekin, Mackay-Whitsunday, and Fitzroy (Fig 1). In Australia, NRM regions are based on catchments or bioregions, and are managed by regional NRM bodies that are responsible for protecting and managing natural resources. In our study, we consider monitoring data collected across four study areas within three of these NRM regions, namely the Russell-Mulgrave and Tully study areas (within the Wet Tropics NRM region), the Burdekin study area (i.e., the Burdekin NRM region), and the Mackay-Whitsunday study area (i.e., the Mackay-Whitsunday NRM region). This study uses inshore water quality data obtained from ambient monitoring using grab samples at fixed locations across these four study areas from 2005 to 2019.

### MMP WQ sampling design: 2005–2014 and 2015–2019

From 2005 to 2014, inshore water quality was monitored at 14 fixed sampling locations across the Wet Tropics, Burdekin, Mackay-Whitsunday, and Fitzroy NRM regions three times per year (wet, early and late dry seasons) by the Australian Institute of Marine Science (AIMS) [34] (Fig 1A). These sampling locations were originally selected to represent measured or presumed gradients in coastal water quality related to river discharge from priority catchments [15, 19, 35]. In this study, we used data from ambient MMP WQ grab sampling across 10 of the 14 locations that were monitored. This included three locations each in the Russell-Mulgrave (RM1, RM7, RM8), the Burdekin (BUR1, BUR2, BUR4), and the Mackay-Whitsunday (WHI0, WHI1, WHI4) region, and one sampling location in the Tully region (TUL3) (Fig 1A and Table 1). These study areas were chosen because monitoring continued at these locations through to 2019, after a change in sampling design implemented in 2015. Five locations (Snapper, WHI0, FTZ1, FTZ2, and FTZ3; Fig 1A) were monitored from 2005 to 2014 but were discontinued in 2014 due to funding constraints.

**Table 1:** Study areas, survey locations and seasonal sampling frequency for the subset of the marine monitoring program used in the power analysis detailed by design i.e., 2005–2014 or 2015–2019.

Design			Sampling frequency	
**2005–2014**	**Study area**	**Locations**	**Wet-season**	**Dry-season**
	**Russell-Mulgrave**	**RM1, RM7, RM8**	**1**	**2**
	**Tully**	**TUL3**	**1**	**2**
	**Burdekin**	**BUR1, BUR2, BUR4**	**1**	**2**
	**Mackay-Whitsunday**	**WHI0, WHI1, WHI4**	**1**	**2**
**2015–2019**				
	**Russell-Mulgrave**	**RM3, RM7, RM8, RM10**	**7**	**3**
		**RM1**	**3**	**2**
	**Tully**	**TUL2, TUL3, TUL5, TUL6,** **TUL8, TUL10**	**7**	**3**
	**Burdekin**	**BUR1, BUR2, BUR4, BUR7, BUR13**	**7**	**2**
		**BUR10**	**6**	**6**
	**Mackay-Whitsunday**	**WHI1, WHI4, WHI5, WHI6, WHI7**	**3**	**2**

The values under sampling frequency refer to the number of sampling visits per year within the wet (November to April) or dry seasons (May to October).

Data from Snapper, FTZ1, FTZ2, and FTZ3 were excluded due to the regions containing these locations not being included in the post-2015 re-design. Data from the WHI0 location were included as they are representative of the pre-2015 sampling design in the Mackay-Whitsunday region. Monitoring at locations in the Fitzroy NRM region recommenced in late 2020, however, due to the gap from 2015 to 2020 these locations were unsuitable and not included in the analyses.

In 2014, GBRMPA undertook a comprehensive review of the overall MMP [36], which included an external assessment of the MMP WQ sampling design [21]. In the context of our study, this review suggested that monitoring of ambient water quality using grab samples and data-logging instruments be retained, but could better meet its objectives by (i) increasing spatial representativeness in high-risk inshore areas (as defined in [37]), and (ii) increasing sampling frequency to investigate potential water quality drivers of other MMP components (i.e., seagrass and coral cover). In 2014, the MMP WQ sampling design was modified based on new knowledge of the spatial patterns of water quality [37–39], recommendations from a statistical analysis of MMP data from 2005–2012 [21], the desire to continue the long-term time series, and trade-offs due to logistical and/or funding constraints.

The current sampling design was implemented in February 2015 and included (i) adding sampling locations in the Cape York, Wet Tropics, Burdekin, and Mackay-Whitsunday NRM regions, and (ii) increasing sampling frequency within regions. Grab sampling across the four study areas of interest occurs now at 22 sampling locations (Fig 1B and Table 1), including most of the original sampling locations (except Snapper, the three Fitzroy locations, and WHI0), allowing for the continuation of the decadal time series.

Sampling frequency within regions increased to between five and twelve times annually and focussed on the Austral summer wet season (twice monthly to monthly) to better characterise this period of higher variability with fewer visits in the drier winter months. Specifically, grab sampling in the Russell-Mulgrave and the Tully study areas was conducted ten times a year and shared equally by AIMS and James Cook University (JCU). For sites in the Burdekin study area (except BUR10) sampling was conducted four times per year by AIMS and five times per year by JCU. Moreover, AIMS conducted grab sampling at BUR10 every month, and in the Whitsunday area (WHI1, WHI4, WHI5, WHI6, WHI7) five times per year.

### Water quality monitoring

The MMP WQ conducts ambient water quality monitoring, including grab sampling during non-event periods (i.e., outside river flooding events), to collect a suite of physical, chemical, and biological water quality analytes at each sampling location [12]. Here, we focus on six water quality analytes, namely total suspended solids (TSS), Secchi disc depth (Secchi), Chlorophyll *a* (Chl-*a*), particulate nitrogen (PN), particulate phosphorus (PP), and nitrate/nitrite (NO_x_). These six analytes are considered relatively robust indicators that integrate several bio-physical processes in the coastal ocean, and water quality guideline values are available for all six of these analytes [40].

From 2015 onwards, the sampling methodologies between AIMS and JCU were generally consistent, however, some institutional differences in analytical methods are described below. At each sampling location, water was collected using Niskin bottles from the sub-surface (~0.5 m below water surface) and bottom (~1 m above the seabed) of the water column between 8:00 and 16:00. From each Niskin bottle, water samples were taken either in duplicate (AIMS) or as single samples with 10% of samples as duplicates (JCU) and processed on-board the vessel as follows. Samples for NO_x_ analysis were immediately filtered (Minisart, pore size 0.45 μm) and the filtrate was frozen until analysis at the laboratory. Concentrations of NO_x_ were determined by segmented flow analysis (Seal AA3 Analyser) at AIMS [41, 42] and JCU (Flow Solution FS3700 OI Analytical segmented flow auto analyser). Samples of Chl-*a* were filtered (Whatman GF/F, pore size 0.7 μm) under vacuum and the filters were stored frozen until analysis. Chl-*a* samples were extracted in 90% acetone, ground, and supernatant fluorescence was read on a fluorometer (Turner 10-AU) at AIMS [43]; JCU samples collected before 2019 were analysed on a Shimadzu UV-1700 spectrophotometer and from 2019 onwards Chl-*a* samples were analysed on a Turner Trilogy 7200–000 fluorometer. PN samples were filtered (Whatman GF/F, pore size 0.7 μm) under vacuum and the filters were stored frozen until analysed by high temperature combustion (Shimadzu TOC-V with Total Nitrogen unit) at AIMS and JCU (from 2018 onwards). Samples of PP were filtered (Whatman GF/F, pore size 0.7 μm) under vacuum and the filters were stored frozen until analysis by persulfate digestion and colorimetric determination on a spectrophotometer at AIMS [44]. The same PP method was followed by JCU from July 2018 onwards; earlier PN and PP values on JCU samples were defined as the difference between total filterable nitrogen/phosphorus (0.45 μm filtered sample) and total nitrogen/phosphorus (unfiltered digested sample) measured on a Flow Solution FS3700 OI Analytical segmented flow auto analyser. Samples of TSS were filtered (polycarbonate, pore size 0.4 μm), rinsed with ultrapure water, and measured gravimetrically at AIMS [45]. The TSS procedure at JCU was similar but used a different filter type (Whatman GF/C, pore size 1.2 μm) in the earlier samples (pre-2019). Secchi depth was determined as the visual limit that a Secchi disc can be seen when cast on the sunlit side of a vessel.

Water quality results from the AIMS laboratory were quality controlled with a series of approaches including procedural blanks, spike recovery in seawater, assessment of analytical accuracy with proficiency testing biannually (Quasimeme, http://www.quasimeme.marlab.ac.uk/about.htm) and annually (the Environmental Nutrient Collaborative Trial program, Australia), and assessment of analytical precision through comparison of duplicate samples. Duplicate sample performance was assessed through the coefficient of variance between duplicates, where this value exceeded 20%, duplicate samples were re-run from spare samples collected at the same location and time. The JCU laboratory also participated in the annual Environmental Nutrient Collaborative Trial program. Every tenth sample at the JCU laboratory was analysed in duplicate and if the mean of the results was >10% then the sample was reanalysed using stored spare samples. After quality control was completed, results were loaded into the AIMS Oracle database. For this study, all available ambient monitoring data were extracted (2005-23-05–2019-07-03) and sampling locations that were not included in our project were excluded. Samples were collected under Marine Park permit numbers G12/35236.1 for AIMS and G15/37587.1 for JCU issued by the Great Barrier Reef Marine Park Authority.

### Preparation of data for power analysis

For each sampling time point at each sampling location, values for each water quality analyte were initially averaged over any duplicate measurements, and subsequently depth-averaged by taking the mean of surface and bottom values, which is the standard for the MMP WQ reporting [46]. Measurements of nitrite and nitrate in the tropical coastal ocean are often below the detection limit (BDL) of analytical instruments, which are reported as half the detection limit (1/2DL) in the MMP WQ dataset. NO_x_ measurements can therefore represent (1) the sum of two BDL measurements, or (2) comprise a BDL measurement and a concentration measurement above the detection limit. For NO_x_, we investigated the implications of imputing BDL values with 1/2DL values on statistical power by comparing results with two other methods (see S1 File and S1 Fig).

**Fig 2:**
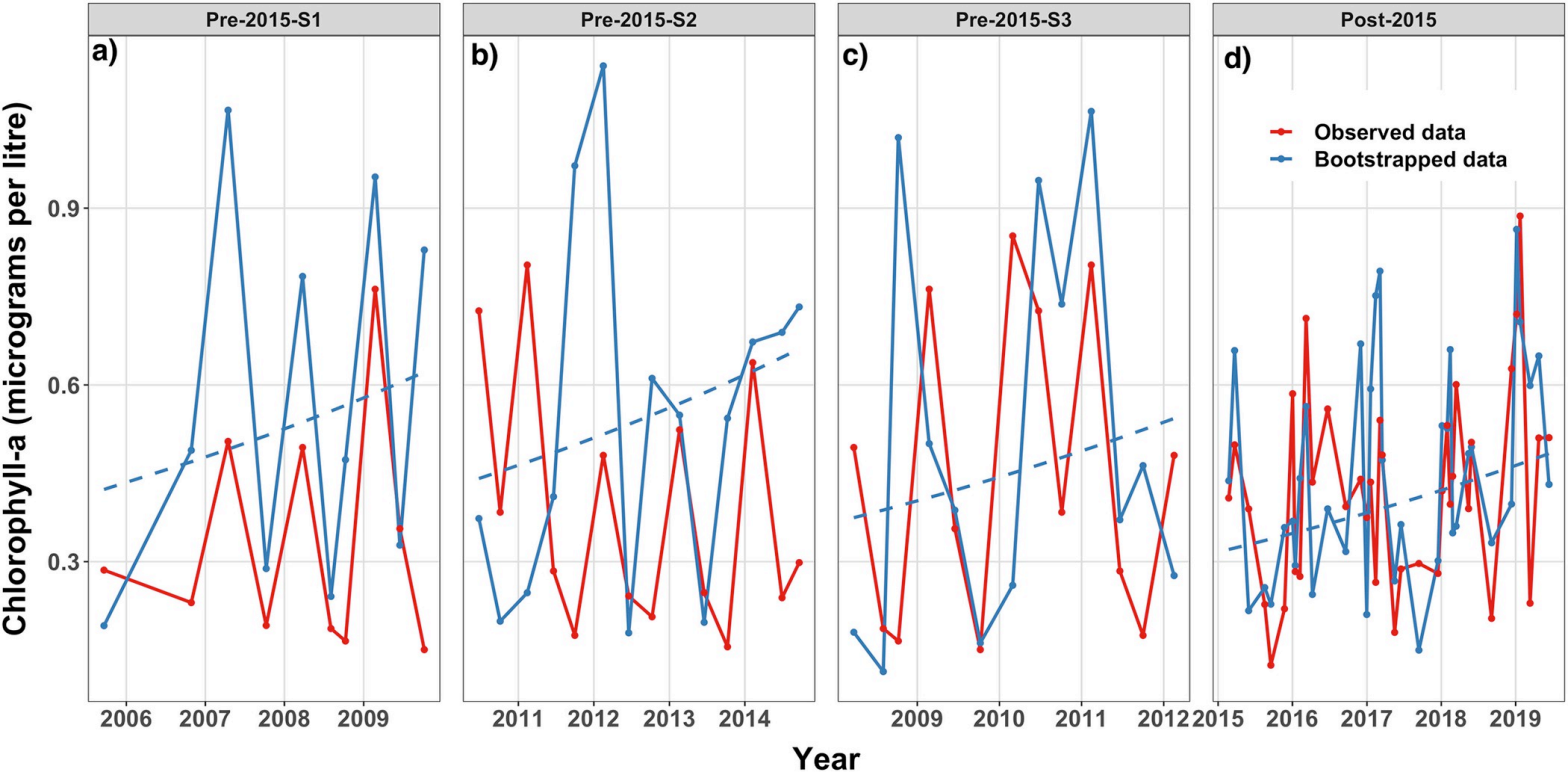
Conceptual representation of one realisation of the bootstrap algorithm. Observed (red) and bootstrapped (blue) data for Chl-*a* concentration (*μ*g L^-1^) taken from a single sampling location (only one of the six Burdekin locations from the regional analysis is shown for clarity) within the Burdekin study area. Panels a) to c) represent the sub-sampled time-series periods for the pre-2015 data, while panel d) depicts the post-2015 data. The blue points and lines represent one replicate of the bootstrap algorithm with a 0.1 year-on-year fractional increase i.e., residuals are resampled from a fit of Model 1 to the observed data and simulated using the expectation of (1) with a new linear term substituted such that the trend (dashed blue line) is increasing at a 0.1 fractional year-on-year change from the intercept term of Model 1.

Water quality data obtained from AIMS and JCU showed differences for some water quality analytes when visualised in time-series plots (see an example in Fig 2). Potential reasons for between-institution variability (AIMS versus JCU) could include temporal differences in sampling times during the year, with JCU conducting more grab sampling during the wet season, which generally has higher variability in water quality data due to greater river discharge [47, 48]. Additionally, AIMS and JCU have used different analytical methods for Chl-*a*, TSS, PP and NO_x_ (detailed above). Regardless, for all six water quality analytes except for NO_x_ there was no *a priori* reason to exclude data from either institution with the observed variability considered a good measure of the natural variation in the ecosystem [33]. For NO_x_, we did not incorporate the JCU data as inter-institutional validation showed differences that could not be attributed solely to measurement timing and were likely related to differences in analytical methods. For the remaining five analytes, we investigated whether there was a mean difference between measurements from AIMS and JCU institutions by including an intercept term indexed by institution in Model 1 below.

### Bootstrap method

We used a bootstrap resampling method to investigate power to detect a linear trend in the measurements of each of the six water quality analytes considered. We propose a simplified parametric version of the model fitted to the MMP data [12, 46]. The simplified model is expected to be straightforward to repeat in future analyses, and in our instance, maintains strong links to the theory surrounding bootstrapping of a regression model, which is weakened by using random-effects or smooth terms [22, 49]. Furthermore, the model is required to be applicable and fixed across two sampling designs with the pre-2015 design being sparser in space and time.

We model the relationship between time and analyte using the following linear regression model with a harmonic seasonal term

$$Y_{i}=\beta_{0}+\beta_{p}+\beta_{s}+\beta_{1}x_{i}+\beta_{2}\;\cos\left(\frac{2\pi {\tilde{x}}_{i}}{T}\right)+\beta_{3}\;\sin\left(\frac{2\pi {\tilde{x}}_{i}}{T}\right)+\epsilon_{i},\;i=1,\ldots ,n,$$ (1)

where the ϵ_*i*_s are uncorrelated with zero means and equal variance *σ*^2^, *n* is the sample size,

*T* = 365.25 is the average length of the calendar year in days, β_0_ is the intercept, β_*p*_ is the effect due to project (p: AIMS or JCU) and, β_*s*_ is the effect on analyte measure due to sampling locations within a region, which vary by region and time-series analysed, β_1_ is the linear trend and β_2_ and β_3_ represent the coefficients characterising the harmonic seasonal component. The data (*x*_*i*_, *y*_*i*_) correspond to the sampling date and the log-transformed analyte measurement on the *i*-th day, respectively. The term ${\tilde{x}}_{i}$ represents the number of days from January 1st of the first sampling year for the seasonal component ensuring that the seasonal curve estimated is relative to January 1st, which aids in interpretation. We log-transform the constituent measurements to improve the positively skewed residual distribution that typically arises in regression analyses where analyte concentration measurements are the dependent variable. This positive skew correction was validated by residual inspection post analysis using Model 1.

The seasonal component is a periodic function of time with a period of one year and models the deviation around the linear trend and has been referred to as the harmonic regression model [50, 51]. Higher order harmonic terms, which allow for more complex seasonal structures, were investigated for pilot analyses but did not improve the model fit (results not shown). The seasonal model is incorporated because concentrations of the six water quality analytes considered here vary with intra-annual seasonal processes such as rainfall and river discharge [47, 48]. Seasonal variation is expected to be detectable at the inter-annual scale due to dry and wet season variation. Initial computation of wet and dry inter-annual averages showed differences for a substantial proportion of the analytes (results not shown). The harmonic model is a simplified parametric version of the generalised additive model smooth term fitted in the MMP reporting [12, 46]. Initial investigations showed that the harmonic model had higher model R^2^ for a majority of the site-analyte combinations than a simple wet-dry dummy variable model, which is a common practice for deseasonalising data [52].

To investigate the exclusion of autocorrelated errors, for all region site-analyte combinations (total number of combinations = 198), we performed the Durbin-Watson test for autocorrelation of residuals [53], from fitting the within site version of Model 1. At an FDR [54] of 5%, only 10 of the 198 site-analyte combinations rejected the null hypothesis of no autocorrelation (of AR1 type) suggesting that autocorrelation is not pervasive across designs and analytes within sites. To investigate the impact of model misspecification due to autocorrelation on inference, which could potentially affect the significance of the trend term within each bootstrap replicate, we investigated the power for the primary analyses using heteroskedasticity and autocorrelation robust standard errors [55] implemented in the sandwich [56] package in R.

To investigate the potential impact of spatial autocorrelation, we visualised residual variograms of residuals of the regional model (Model 1) in the gstat [57] R package. We further performed the permutation test for Moran’s I statistic in the spdep [58] package in R. No general evidence for spatial autocorrelation in the residuals was observed from these analyses (results not shown). Clustered sandwich estimators [59] were also investigated to adjust inference in the trend parameter for the potential that errors are spatially correlated within (but not between) sites in the R package sandwich.

For each constituent and subset of the total time-series we estimated the parameters of Model 1 and implemented a bootstrap resampling algorithm that computes the power for a set of target trend slope coefficients defined as the fractional year-on-year change (*δ*) (that is, 100*δ* is the percentage change) in analyte values (see S2 File for further algorithm details).

### Power change between pre-2015 and post-2015 sampling designs

To investigate whether the sampling re-design influenced the power to detect a linear trend in measurements of individual water quality analytes, we split the data time series into four time periods: data with dates greater than 2005-09-18 and less than 2009-12-30 (pre-2015-S1); greater than or equal to 2010-06-15 and less than 2015-01-01 (pre-2015-S2); between 2008-02-01 and 2012-05-09 (pre-2015-S3); and after 2015-01-01 (post-2015). Each of these three pre-2015 time periods span approximately 1,550 days, which is the length of the data available for the post-2015 sampling period. This was done to allow for a valid comparison between the pre-2015 and post-2015 sampling regimes using equal sampling periods (~5 years). The first two pre-2015 time periods (pre-2015-S1, pre-2015-S2) were chosen as a natural division of the ten-year pre-2015 study period. The third pre-2015 period (pre-2015-S3) was taken from the intermediate years to average over any potential processes unique to the first two.

For each period, we estimated the parameters over the individual study area (i.e., all sampling locations included) using the linear model described in Model 1 for each of the six water quality analytes and for each of the four study areas. We used Algorithm 1 to estimate the power to detect a linear trend with *δ* = (−0.2, −0.19, …, 0.19, 0.2) and *R* = 1,000. The bootstrap process is conceptualised in Fig 2 for one bootstrap iteration using a fractional year-on-year increase of 0.1 and data for Chl-*a* from a single sampling location (BUR1), which is a component of the regional bootstrap analysis in the Burdekin area.

### Power change for increasing sampling locations and sampling frequency

To investigate whether adding sampling locations or increasing per-year sampling frequency contributed to the greatest relative improvement in power, we partitioned the post-2015 data into the following sets: 1) we sub-sampled the post-2015 data to only those sampling locations used in the pre-2015 sampling regime and a similar within-year sampling density to pre-2015, which is three samples per year (baseline/null data); 2) we retained the same within-year sampling density (three samples per year) as in 1) but added in the extra sampling locations for each study area to the post-2015 data (i.e., effect of additional sampling locations); and 3) we retained the pre-2015 sampling locations but increased the sampling density to the post-2015 data (i.e., effect of additional samples). For comparison we also incorporated the results from all data after 2015.

Using these four data sets, we investigated the power to detect a linear trend using Model 1 for five of the six priority water quality analytes (excluding NO_x_) and for each of the four study areas. NO_x_ was excluded from this analysis as the removal of the JCU data limits the capacity to increase the sampling density to be comparable with the other analytes in assessing the effect of additional samples. Note that for the Mackay-Whitsunday area the sampling frequency only increases from three to five and thus a smaller power increase is expected relative to other regions. To estimate power, we used the bootstrap algorithm with _*δ*_ = (−0.2, −0.19, …, 0.19, 0.2) and *R* = 1,000.

## Results

### Power change between pre-2015 and post-2015 sampling designs

The bootstrap resampling results refer to the power to detect a linear trend in a regional scale linear model (Model 1) applied to each water quality analyte (i.e., TSS, Secchi, Chl-*a*, PN, PP, and NO_x_) in each of the four study areas (i.e., Russell-Mulgrave, Tully, Burdekin, and Mackay-Whitsunday). Parameter estimates and significant terms at a 5% false discovery rate [54] can be viewed for each model in S1 Table. Power curves were generated for each of the six water quality analytes and each of the four time series periods (S3 Fig).

To reduce the complexity of interpreting these power curves we averaged the power values over δ = (−0.1, 0.1), which are approximately equal for most analytes but are different for some (e.g., NO_x_; S3 Fig). For all analytes, except NO_x_, a fractional value of 0.1 examines the 5-year trend and power to detect a decline (exceedance of the 10th percentile of the post-2015 analyte measurements) or increase (exceedance from the 90th percentile of the post-2015 analyte measurements) in analyte values (see example calculations in S4 Fig). In the case of NO_x_, a 12-year trend is examined. The average (over sampling locations in each study area) computed time to exceed the 10^th^ and 90^th^ percentiles of the post-2015 data for each analyte-study area combination is presented in S5 Fig and S2 Table. Furthermore, we averaged the power at a 0.1 fractional change over the three pre-2015 time-series periods for comparison with the post-2015 results (see S6 Fig for results from each scenario). These summaries show that at a 0.1 fractional change the yearly mean for most analytes will exceed the tails of the current distribution of values within four to six years and in the case of NO_x_, 12 to 16 years (S5 Fig).

**Fig 3:**
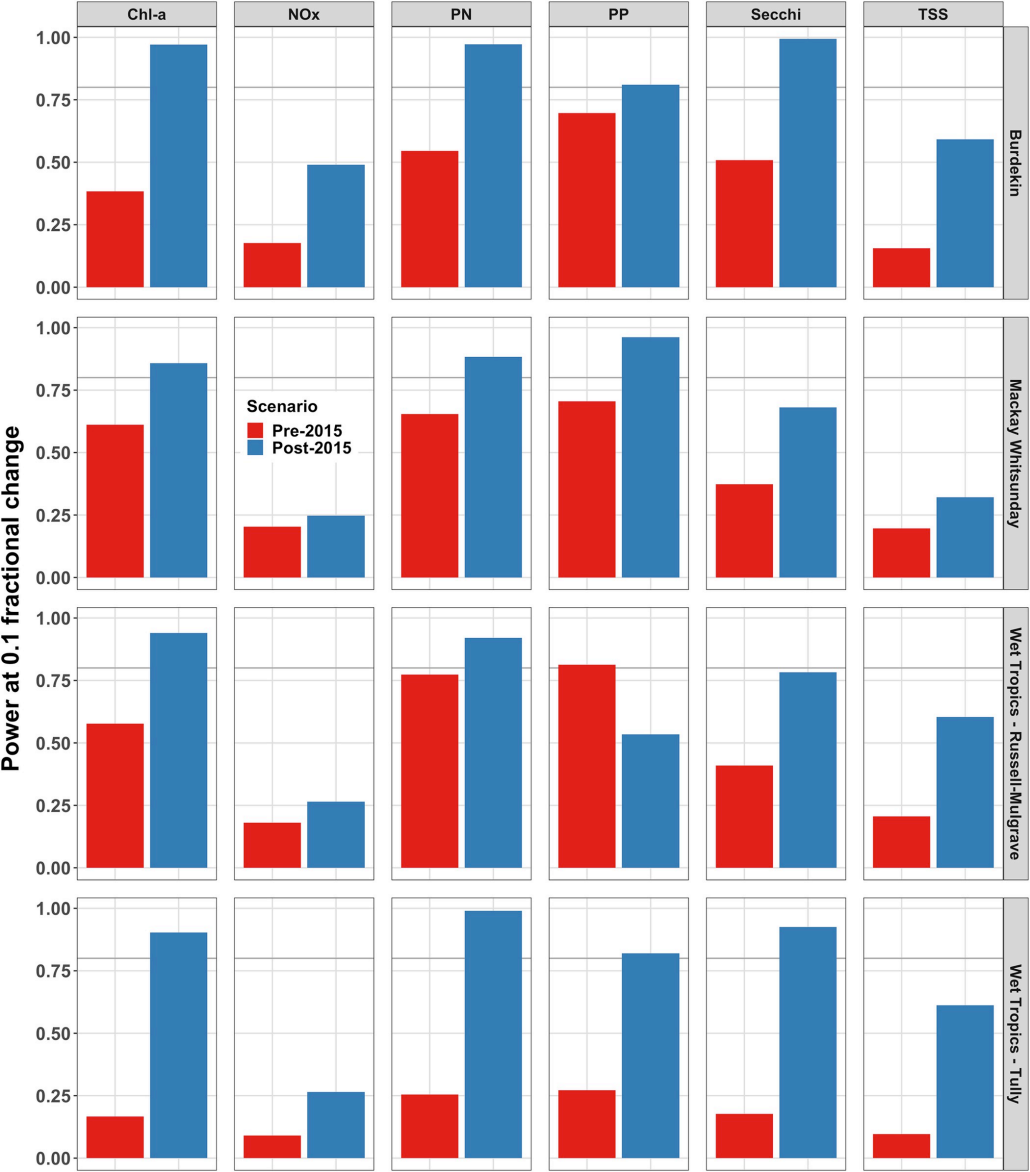
Power at 0.1 fractional change for six water quality analytes for pre- and post-2015 sampling designs across four study areas. The height of each bar within each panel is the average of the two power values for δ = (−0.1, 0.1) i.e., an increasing and decreasing year-on-year fractional change of 0.1 in analyte values. The pre-2015 (red) and post-2016 (blue) values in each panel represents a further average over three time periods (see S3 and S6 Figs). Darker grey horizontal line represents 80% power. The columns are presented for Chlorophyll *a* (Chl-*a*), nitrate/nitrite (NO_x_), particulate nitrogen (PN), particulate phosphorus (PP), Secchi depth (Secchi), and total suspended solids (TSS), for the Burdekin, Mackay-Whitsunday, Russell-Mulgrave and Tully study areas.

The power analysis showed that for 23 of the 24 analyte-study area combinations the post-2015 data had higher power to detect a 0.1 fractional linear change in analyte measurements over a five-year period (Fig 3). Overall, we observed an average power increase of ≈ 34% across all analyte-study area combinations. Increases in the absolute value of power to detect a 0.1 fractional linear change ranged from 4.5% for NO_x_ in the Mackay-Whitsunday study area to 75% (15% to 90%) for Secchi depth in the Tully study area. The power to detect a 0.1 fractional change exceeded 80% for 13 of the 24 analyte-study area combinations in the post-2015 scenario that previously had power less than 80%.

The one combination that did not show increased power in the post-2015 data was PP in the Russell-Mulgrave study area, which had approximately 30% more power in the pre-2015 data. In this case, the raw PP data (averaged over depth and measure replicates) showed substantial differences in variation between AIMS and JCU measurements, with reduced samples for JCU from one of the sampling locations (S7 Fig). For comparison, the AIMS and JCU measurements for Chl-*a* in the Russell-Mulgrave study area showed higher consistency in both value and variability (S8 Fig).

Substantial increases in power were observed for the post-2015 data in the Tully study area, which is a likely result of the increase in sampling locations from one to six and the increased sampling frequency (see S9 Fig for a representative example of the increase in sampling for Secchi depth). For example, the power to detect a 0.1 fractional change for Secchi depth increased by a factor of four to approximately 90% after the sampling re-design in the Tully area. S10 and S11 Figs showed no change in the primary inference summarised above using the heteroscedastic and autocorrelation robust or cluster robust standard errors, which implies that Model 1 is reasonably specified.

### Power change for increasing sampling locations and sampling frequency

**Fig 4:**
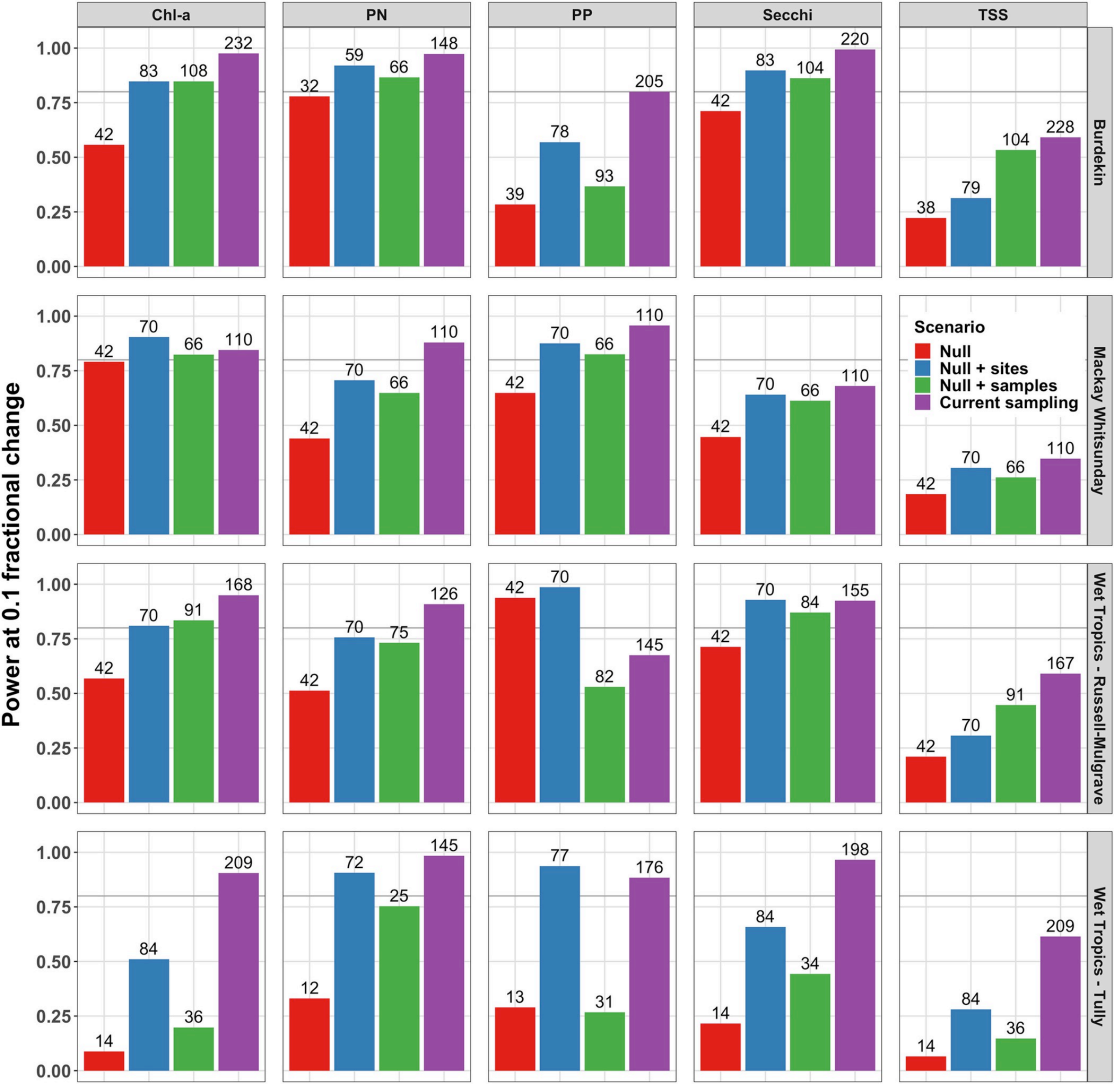
Power at 0.1 fractional change for six water quality analytes investigating the change in power due to increasing sampling frequency or adding sampling locations. The height of each bar within each panel is the average of the two power values for δ = (−0.1, 0.1) for each of the subsamples of the post-2015 data namely: all data after 2015-01-01 data (current sampling (purple)); down sample the post-2015 data to those sites used in the pre-2015 sampling regime and a similar within year sampling density to pre-2015 (null (red)); retain the same within year sampling frequency but add in the extra sites for each area to the post-2015 data (null + sites (blue)); and retain the pre-2015 sites but increase the sampling density to the post-2015 data (null + samples (green)). The number above each bar corresponds to the sample size of the down sampled data set that was used to estimate Model 1 and subsequently in the bootstrap power analysis. Darker grey horizontal line represents 80% power. The columns are presented for Chlorophyll a (Chl-a), nitrate/nitrite (NOx), particulate nitrogen (PN), particulate phosphorus (PP), Secchi depth (Secchi), and total suspended solids (TSS), for the Burdekin, Mackay-Whitsunday, Russell-Mulgrave and Tully study areas.

Power curves were generated for each of the analyte-study area combinations across the down sampled post-2015 data scenarios. The power curves were summarised as the average power values over _*δ*_ = (−0.1, 0.1) (Figs 4 and S12). The sampling location versus sampling frequency analysis showed that the retention of the pre-2015 sampling regime gave substantially reduced power relative to the current post-2015 sampling design at a 0.1 fractional change for all but one analyte-study area combination. The exception was again PP in the Russell-Mulgrave area, which showed increased power with the addition of sampling locations but reduced power when the within-year sampling frequency was increased. The addition of sampling locations always increased power relative to the null scenario with varying degrees of improvement across analyte-study area combinations. Similarly, increasing sampling frequency always increased power relative to the null scenarios except for PP in the Russell-Mulgrave and Tully study areas. For TSS in the Burdekin study area, and TSS and Chl-*a* in the Russell-Mulgrave study area, increasing the sampling frequency equalled or increased power compared to adding sampling locations. Overall, both the addition of sampling locations and increasing sampling frequency increased the power to detect change relative to the null scenario. However, the addition of sampling locations gave the greatest increase in power to detect change on average per sample size.

## Discussion

Sampling designs are crucial to ensuring that measurements in monitoring programs are capable of evaluating progress toward clearly stated management objectives [1–4]. Here, we used a 15-year monitoring dataset to investigate the effect of sampling re-design on the statistical power to detect trends in marine water quality. We performed a comprehensive bootstrap resampling analysis to assess the power for six water quality analytes at four separate study areas in the GBR Marine Park. We found that the sampling re-design increased power to detect trends in 23 of the 24 analyte-study area combinations. Specifically, our results showed an average increase in power of 34% at an increasing or decreasing 0.1 fractional change in year-on-year analyte values. Furthermore, we demonstrated that this increase in power is attributed more to the addition of sampling locations (i.e., spatial coverage) than increasing the sampling frequency (i.e., temporal coverage). Therefore, the sampling re-design has substantially increased the capacity of the MMP WQ to detect temporal trends in inshore marine water quality, which is a component of its primary objective [18].

### Sampling re-design improves power

To our knowledge, this is one of the only studies to have utilised long-term monitoring data from both before and after a sampling program re-design to determine if the intervention has improved statistical power. The study further required knowledge of the purpose and intervention implementation, consistency in measurement practices, and the resources to complete an assessment of the impact. Modern examples of power analysis in ecological monitoring typically assess the capacity of a current monitoring program to answer key questions aligned with its objectives [60–63] and/or simulate prospective optimal sampling re-designs that integrate new methodologies (analytical or experimental) and constraints on resources [8, 64, 65]. O’Hare et al. [6] simulated the effect of network re-design on the statistical power to detect long-term trends across a national monitoring network of three key ecological indicators. O’Hare et al. [6] highlighted that one caveat of simulated re-designs is that the process assumes that the trends and structure of noise in future monitoring data follow similar patterns to the pilot data. Our study is not subject to this caveat because the re-design data are observed. For example, a simulation approach would not have integrated the increase in total data variance related to increased sampling during the wet season, which was observed in the post-2015 data.

Although our study showed that the sampling re-design substantially increased power to detect trends, 11 combinations did not exceed 80% power in the re-design. NOx and TSS did not exceed 80% power for any area-analyte combination and showed the lowest power (< 20%) on average in the pre-2015 sampling design. Likely reasons for these systematically low baseline power values for NOx and TSS include NOx being the sum of nitrate and nitrate measurements, which leads to many more below the detection limit values. These measurements require imputation, which increases trend uncertainty and lowers power. Across all sites, on average, TSS showed the highest variability of all analytes (SD of mean adjusted values of 3.25 relative to 0.372 for Chl-a) and the highest between-site variability within study areas. This increased variation limits power as the sample size needs to be larger to compensate for this higher variability to achieve similar power. Darnell et al. [66] also observed low levels of power to detect a trend in TSS and attributed it most likely to climate signal variability and measurement uncertainty.

Darnell et al. [66] further examined the power to detect trends in total suspended sediment loads in the Burdekin and Tully areas of the GBR commensurate with the Reef Plan’s goal of a 20% reduction in sediment by 2020. Darnell et al. [66] used a simulation to show that the end of catchment monitoring programs had low statistical power to obtain the 2020 goal and that a 10% year-on-year change over 20 years was required to reach 80% power. A key difference of our study from that of Darnell et al. [66], and other studies of monitoring design and power, is that we had the benefit of an observed post-re-design data set. These data allowed for the utilisation of bootstrap resampling on both the pre- and post-2015 data sets. The bootstrap resampling of the residuals algorithm offers an intuitive integration of the hypothesised effect sizes with the observed model variation. The bootstrap algorithm improves the estimation of the trend effect uncertainty, and subsequent estimates of power, when normal-theory distributions of the estimators are inaccurate [22]. Reflections on this study should consider that the power analysis results are conditional on the model fitted. For example, if a more complex model explained more of the total data variability then this may increase the power to detect trends. As our study was comparative, we did not explore more complex models in detail, however, models including spatial components would be a likely next step [8].

### Fractional year-on-year change in analyte concentration

In our study we performed summary comparisons of the two sampling designs at a 0.1 fractional year-on-year change, because the mean value of most analytes would exceed the 10th or 90th percentile of the post-2015 data within four to six years on average across sampling locations within a study area. Therefore, the 0.1 fractional year-on-year change represents a forward projection where the current ecological state moves to a new state within five years (on average). Most previous research on changes in coastal water quality has focused on nutrient inputs (nutrient loading) to the system, typically in the context of management interventions. Only a few studies, however, have reported measured improvements in river pollutant loads and coastal water quality following catchment management [67]. Recently, Lefcheck et al. [68] reported that sustained management actions had reduced measured nitrogen concentrations in Chesapeake Bay by 23% and phosphorus concentrations by 8% since 1984. Riemann et al. [69] described a reduction in Danish coastal waters of flow-weighted concentrations of dissolved inorganic nitrogen, total nitrogen (TN), dissolved inorganic phosphorus, and total phosphorus, with the TN loading decreasing by ∼50% relative to pre-action values within 10–15 years. From an increasing trend perspective, Harding et al. [70] analysed trends in periods (1945–1980) of eutrophication in Chesapeake Bay that showed a doubling of TN and NO_x_ loading (increases of >120% and 90%, respectively). In a global meta-analysis of recovery of lakes and coastal marine ecosystems from eutrophication, McCrackin et al. [71] reported that on average coastal marine areas achieved 24% of their baseline conditions decades after the cessation or partial reduction of nutrient loading.

Within the MMP WQ, annual mean or median values of water quality analytes are compared to GBR water quality guideline values to assess condition [40]. Linking fractional year-on-year change to these guideline values was not feasible given the variability in guideline values by sample location and region, coupled with many sample locations within a study area having a yearly mean or median close to their guideline values [12]. The validation of guideline values was not an objective of this study, so we instead used a standard 0.1 fractional year-on-year change to allow comparison in study design at the regional and whole-of-program scale. Although major summaries are made at a 0.1 fractional year-on-year change, comparisons are possible for any value between -0.2 and 0.2 through inspection of supplementary power results. We assumed that this year-on-year fractional change would be the same for each of the sampling locations with different initial starting values. Model 1 does not consider that within sampling location trends could change at different rates, with the study area estimate averaging the within sampling location trends over the study area. Weiser et al. [72] investigated the implications of this averaging and found higher power is achieved when grouped sites have similar relationships. Within-sampling location power analyses could resolve this and would be simple to implement in future studies using the methodology presented.

### Particulate phosphorus in the Russell-Mulgrave region: The one exception

The power to detect change decreased in the PP data in the Russell-Mulgrave study area, presenting an opportunity to understand its potential root cause. The raw post-2015 data showed substantially increased variation, with approximately six times the variation in PP measurements compared to the pre-2015 data (S7 Fig). Under a set of simplified model assumptions, the multiple regression power calculation depends on the sample size and the model *R*^2^, which summarises the proportion of total data variation accounted for by the linear predictor [73]. Although the variation in the linear predictor for PP explained three times the variation in the post-2015 model when compared with the pre-2015 linear predictor, the increase in total data variance reduced the model R^2^. Substantial data investigation prior and post power analysis suggests a potential for increased variation in PP to be derived from the differences in analytical methods for PP concentration measurement between AIMS and JCU, the sampling by JCU in higher variability wet-season, and the addition of inshore sites, which may be more responsive to river discharge. The JCU lab method for PP uses the difference between the total (unfiltered) and filtered sample. The final result has the uncertainties in the analysis of both results (i.e., the filtered and unfiltered sample) and hence the precision of the final result may be less than via a direct measure. Further evidence for the high-variation observations limiting power was observed in the increasing sampling locations versus sampling frequency analysis, where the ‘addition of locations’ scenario had substantially higher power relative to the full post-2015 data. In this analysis, the high-variance measurements are excluded because the data are sub-sampled to the pre-2015 sampling frequencies when monitoring was performed 2 out of 3 times in the less variable dry season. These high-variance measurements are also present in the Tully and Burdekin regions at similar sampling instances to those observed in the Russell-Mulgrave (see online results tool in Code Availability section). We observed that the power decreased for PP in the Tully area when we removed these JCU observations (seen in the ’Null + sites’ results in Fig 4), indicating that these high variability JCU measurements influenced power across study areas. The increase from one to six sites was substantial enough to increase power for the Tully region in the pre- versus post-sampling design analysis. This anomaly highlights the importance of standardising field and laboratory methodologies in monitoring programs to reduce variation and maximise statistical power.

### The importance of spatial versus temporal coverage

The increase in power to detect change following the sampling re-design can be attributed more to the addition of sampling locations (i.e., spatial coverage) than increasing the sampling frequency (i.e., temporal coverage). Spatial variability could be related to differences in, and distances from, land-based inputs [74], as well as oceanic drivers including resuspension, transport, and shelf biogeochemical processes and metabolism [75, 76]. Andersen and Steidl [77] through a power simulation study of terrestrial fauna observed that for the same sampling effort across space and time, the highest power to detect changes in abundance was achieved by maximising the number of sites. Importantly, the ‘addition of sampling locations’ analysis has the caveat that the sampling frequency is reduced to the pre-2015 rate when monitoring was performed 2 out of 3 times in a less variable period of the year (i.e., the dry season). This reduces total variability in the data and is a likely contributor to the increased power of the ‘addition of sampling locations’ scenario relative to the ‘increased sampling rate’ scenario. Across all analyte-study area combinations, the median post-2015 data variance increased by a factor of 3.7 with only Secchi depth in the Mackay-Whitsunday having a value less than unity (0.541). Contributing factors to this increased variation likely include the increased spatial coverage of the new design, which incorporated new sites with higher variability (such as sites near river mouths), and an increased frequency of monitoring in the higher-variability wet season period (3 times dry season and 7 times wet season). This capturing of the increased variation limits the power in the post-2015 data relative to the pre-2015 data. It is interesting that the increase in sample size has been substantial enough to compensate for this increase in variation and has led to a more powerful sampling design for detecting temporal trends for nearly all analyte-study area combinations.

### Implications for monitoring and management

The findings of this study demonstrate that the sampling re-design in 2015 has increased the statistical power to meet the MMP WQ’s objective of detecting trends in inshore ambient water quality. These findings provide confidence in the current MMP WQ sampling design, and a similar sampling design recently implemented in the Fitzroy NRM region. Our analysis highlights the benefits and drawbacks of using two different laboratories across a monitoring program. Indeed, using two laboratories can increase confidence in the dataset with added QA/QC components such as inter-laboratory comparisons to demonstrate the veracity and replication of the datasets. However, where differences in the laboratory methods are apparent, having separate laboratories can create added uncertainty and reduce power, as observed for PP in our analysis. Although trend estimation was performed in our analysis, trends in water quality analytes are formally reported on and the implications against management guideline values are discussed in the recent MMP WQ program reporting [46]. Future analyses should focus on further optimising these sampling designs to reliably detect trends within timeframes where improvements in inshore water quality can be expected to occur based on management activities in GBR catchments [67, 68]. Furthermore, while the increased sampling frequency did not contribute as much to the improved power to detect change compared to the increased sampling locations, it did allow for a more comprehensive assessment of the within-year analyte variation. Namely, increased sampling during the wet season allowed the program to better characterise this period of higher variability, which is a natural feature of tropical coastal environments. This monitoring is valuable in linking pollutant concentrations to end-of-catchment loads [78, 79], another overarching objective of the MMP WQ [18]. Moreover, increased within-year sampling at locations where other components of the inshore environments are monitored, such as seagrass condition [74] and coral health [80], strengthen investigations into potential water quality drivers of these components [21].

## Conclusions

In summary, we have used a real-world example of a well-established monitoring program, namely the GBR MMP WQ, to investigate the effect of sampling re-design on the statistical power to detect trends in marine water quality. Using a bootstrap resampling algorithm, we performed a comprehensive assessment of power for six water quality analytes at four separate study areas in the GBR Marine Park and showed that the sampling re-design increased power to detect trends in 23 of the 24 analyte-study area combinations. On average per unit sampling effort, this increase in power was attributed more to the addition of sampling locations than increasing the sampling frequency. However, the increased sampling frequency allowed for a more comprehensive assessment of the within-year analyte variation, which is highly valuable information for future sampling designs and other components of the MMP. The sampling re-design has substantially increased the capacity of the MMP WQ to detect temporal trends in inshore marine water quality, which is a component of its primary objective [18]. The evaluation of the MMP WQ to meet its objectives following the sampling re-design has closed an iteration of the adaptive monitoring and management cycle of review, implementation, and re-evaluation to identify potential program improvements. The results of this study provide confidence that the sampling re-design has improved the probability of detecting trends in inshore water quality, including changes that may be driven by reduced end-of-catchment pollutant loads because of catchment management actions.

## Supporting information

S1 File Investigating implications for power due to imputation methods for below the detection limit values for NO_x_. (PDF)

S2 File Detailed bootstrap power algorithm. (PDF)

S1 Fig Bootstrap power results for below detection limit (BDL) imputation investigation for NO_x_ constituent in the Burdekin study area. (PDF)

S2 Fig Time series of Chlorophyll *a* (Chl-*a*) concentrations for the five sampling locations in the Burdekin study area. (PDF)

S3 Fig Power curves from pre- and post-2015 sampling design comparisons for six water quality analytes across four sampling areas. (PDF)

S4 Fig Time to exceedance of 10th and 90th percentile for Chl-*a* for six sampling locations in the Burdekin study area. (PDF)

S5 Fig Time (in years) for linear trend to exceed the 10th and 90th percentile for six water quality analytes across four sampling areas at a 0.20 or 0.10 fractional year-on-year change. (PDF)

S6 Fig Power at 0.1 fractional change for six water quality analytes for pre- and post-2015 sampling regimes across four study areas. (PDF)

S7 Fig Time series of particulate phosphorus (PP) concentrations for the five sampling locations in the Russell-Mulgrave study area, within the wet tropics natural resource management region. (PDF)

S8 Fig Time series of Chlorophyll *a* (Chl-*a*) concentrations for the five sampling locations in the Russell-Mulgrave study area, within the wet tropics natural resource management region. (PDF)

S9 Fig Time series of Secchi depth for the six sampling locations in the Tully study area, within the wet tropics natural resource management region. (PDF)

S10 Fig Power at 0.1 fractional change using heteroskedasticity and autocorrelation robust standard errors for six water quality analytes for pre- and post-2015 sampling regimes across four study areas. (PDF)

S11 Fig Power at 0.1 fractional change for six water quality analytes using cluster robust standard errors for pre- and post-2015 sampling regimes across four study areas. (PDF)

S12 Fig Power curves for investigating the change in power due to increasing sampling frequency or adding sampling locations. (PDF)

S1 Table Parameter estimates for fitted model for each of the pre-2015 and post-2015 data scenarios. Table columns include analyte, Pre-2015 S1, S2, S3 and Post-2015, study area, parameter, estimate of the parameter, standard error of the estimate, t-value, p-value, the transformed FDR p-value, logical for parameter significance at 5% FDR and parameter group of intercept, site, project, trend and seasonal terms.
(XLS)

S2 Table Time in years to exceed the 10th or 90th quantile of the post-2015 data for each constituent and study area. Table columns include REGION—four study areas, DELTA—fractional year-on-year change, ANALYTE—analyte analysed, MEAN_TIME_Q10—time in years for projected linear trend to go below the 10th percentile of the post-2015 data, MEAN_TIME_Q90—time in years for projected linear trend to go above the 90th percentile of the post-2015 data.
(XLS)
