# Supplementary material for: Sampling re-design increases power to detect change in the Great Barrier Reef’s inshore water quality
Source: PLoS One. 2022 Jul 28;17(7):e0271930. doi: 10.1371/journal.pone.0271930 (PMC9333274; doi:10.1371/journal.pone.0271930)
Supplement: S1 File — (PDF) [file pone.0271930.s001.pdf]

S1 Note

Investigating implications for power due to imputation methods for below the detection limit values for NO_x

We assessed the implications for Model 1 and subsequent power analysis in relation to the imputation of BDL measurements using three approaches. Specifically, we compared results from using (1) 1/2DL , (2) half the minimum, and (3) a method for imputing BDL measurements using the robust Regression on Order Statistics (rROS) method (Helsel, 2005). The latter approach was implemented using the EnvStats (Millard, 2014) R package to estimate the mean and variance of the lognormal distribution for the non-BDL nitrite and nitrate measurements. Nitrite and nitrate measurement BDL values were imputed with random values drawn from a log-normal distribution (mean and variance estimated from rROS) truncated at the detection limit specific to each observation i.e., to incorporate variability in detection limits.

Regional scale analyses using model Equation (1) for NO_x measurements pre-2015 and post-2015 from the Burdekin were performed. Using the residuals from the estimated models, bootstrap power analyses were performed with $R = 500$ and $\delta = (-0.2, -0.18, \ldots, 0.18, 0.2)$ for data processed using the three methods. Power for imputation using 1/2DL gave the highest power values, with the second and third approaches giving similar power across the two scenarios (S1 Fig). Quantile residual plots for each of the estimated models indicated a good fit to the data. Therefore, for all analyses, we constructed NO_x measurements from nitrite and nitrate values that were imputed as 1/2DL and averaged as described above.

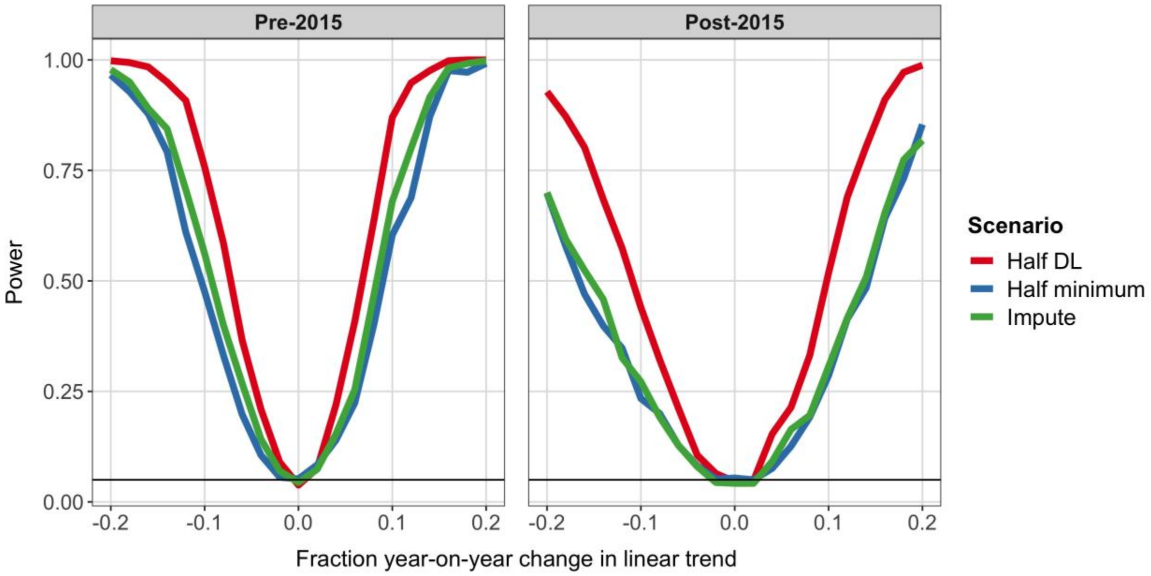

S1 Fig. Bootstrap power results for below detection limit (BDL) imputation investigation for NO_x constituent in the Burdekin study area. Power curves for comparing method of imputing values of below detection limit values for nitrite and nitrate prior to summation to form the NO_x analyte. All data before 2015-01-01 were used in the pre-2015 analysis and for the post-2015 analysis. No sub setting of the pre-2015 data were performed for this analysis as we were interested in comparing the imputation methods rather than comparing power between sampling regimes. Power in these plots refers to the linear trend component and is from a regional analysis of NO_x from the Burdekin study area. Other study area showed similar differences in power curves between BDL imputation methods. The imputation scenarios are: impute the BDL with half the detection limit (Half DL): impute the BDL values with half the minimum value over all measurements (Half minimum); and impute the BDLs using sampled values from a log-normal distribution with mean and variance parameters estimated using the rROS method (Impute). The x-axis corresponds to the simulated fractional year-on-year changes that ranged from $\delta = (-0.2, -0.18, \ldots, 0.18, 0.2)$. Darker grey horizontal line represents 80% power.

References

Helsel, D. R. (2005) *Nondetects and data analysis. Statistics for censored environmental data.* Wiley-Interscience.

Millard, S. P. (2014) ‘E nv S tats, an R Package for Environmental Statistics’, *Wiley StatsRef: Statistics Reference Online*.
