# Supplementary material for: Sampling re-design increases power to detect change in the Great Barrier Reef’s inshore water quality
Source: PLoS One. 2022 Jul 28;17(7):e0271930. doi: 10.1371/journal.pone.0271930 (PMC9333274; doi:10.1371/journal.pone.0271930)
Supplement: S2 File — (PDF) [file pone.0271930.s002.pdf]

S2 Note

For each constituent and subset of the total time-series we estimate the parameters of Model 1 by ordinary least squares. The raw residuals from this model are $e_i = y_i - \hat{\mu}_i$ and the estimated mean values $\hat{\mu}_i = \widehat{\beta_0} + \widehat{\beta_p} + \widehat{\beta_s} + \widehat{\beta_1} x_i + \widehat{\beta_2} \cos\left(\frac{2\pi\tilde{x}_i}{T}\right) + \widehat{\beta_3} \sin\left(\frac{2\pi\tilde{x}_i}{T}\right)$. Following Davison and Hinkley, (1997) we implement a bootstrap resampling algorithm that uses the modified residual value m_i for sample i and is defined as $m_i = \frac{e_i}{\sqrt{1-h_i}}$, where h_i is the leverage for sample i. The modified residuals account for individual points with high leverages and is the recommendation of Davison and Hinkley, (1997). We define $\bar{m}$ to be the sample average of the modified residuals. The modified Furthermore, we introduce the term $\beta_{1\delta}$, which is the slope coefficient that corresponds to a fractional year-on-year linear change in analyte concentration. We define δ as the target fractional change, (that is, 100δ is the percentage change), which is specified. Therefore, the slope coefficient is $\beta_{1\delta} = \log(1-\delta)/T$ to ensure that the change on the original scale corresponds to δ year-on-year. Then, for each δ and model we compute the power i.e., the probability we reject the null hypothesis that the regression slope coefficient is greater or less than zero when it is in fact non-zero. Supplementary Algorithm 1 (below) details the algorithm steps.

S1 Algorithm - Bootstrap power algorithm

For $r = 1, \ldots, R$ replicates perform

1. For $i = 1, \ldots, n$,

   a) set $x_i^* = x_i$;

   b) randomly sample ϵ_i^* from the modified residuals $m_1 - \bar{m}, \ldots, m_n - \bar{m}$;

   c) set $y_i^* = \widehat{\beta_0} + \widehat{\beta_p} + \widehat{\beta_s} + \beta_{1,\delta} x_i + \widehat{\beta_2} \cos\left(\frac{2\pi\tilde{x}_i}{T}\right) + \widehat{\beta_3} \sin\left(\frac{2\pi\tilde{x}_i}{T}\right) + \epsilon_i^*$.

2. Fit least squares regression to $(x_1^*, y_1^*), \ldots, (x_n^*, y_n^*)$ giving

   estimates $\widehat{\beta_{0,r}^*}$, $\widehat{\beta_{p,r}}$, $\widehat{\beta_{s,r}}$, $\widehat{\beta_{1,\delta,r}}$, $\widehat{\beta_{2,r}}$, $\widehat{\beta_{3,r}}$ and s_r^{2*} (error variance estimate).

Compute power $B(\beta_{1,\delta}) = \frac{\sum_{r=1}^{R} I[|t_r^*| \geq \tau_{\alpha/2,(n-2)}]}{R}$, where $t_r^* = \frac{\widehat{\beta_{1\delta,r}}}{SE(\widehat{\beta_{1\delta,r}})}$, $\mathbb{P}(-\tau_{\alpha/2} < \mathcal{T} < \tau_{\alpha/2}) = 1 - \alpha$, where $\mathcal{T}$ has a Student's t-distribution with $n - 2$ degrees of freedom, we set $\alpha = 0.05$, $SE(\widehat{\beta_{1\delta,r}})$ is the estimated standard error of the regression slope coefficient for bootstrap replicate r, and *I* is the indicator function.

References

Davison AC, Hinkley DV. Bootstrap methods and their application. New York: Cambridge University Press; 1997.
