# Supplementary material for: Sampling re-design increases power to detect change in the Great Barrier Reef’s inshore water quality
Source: PLoS One. 2022 Jul 28;17(7):e0271930. doi: 10.1371/journal.pone.0271930 (PMC9333274; doi:10.1371/journal.pone.0271930)
Supplement: S1 Fig — (PDF) [file pone.0271930.s003.pdf]

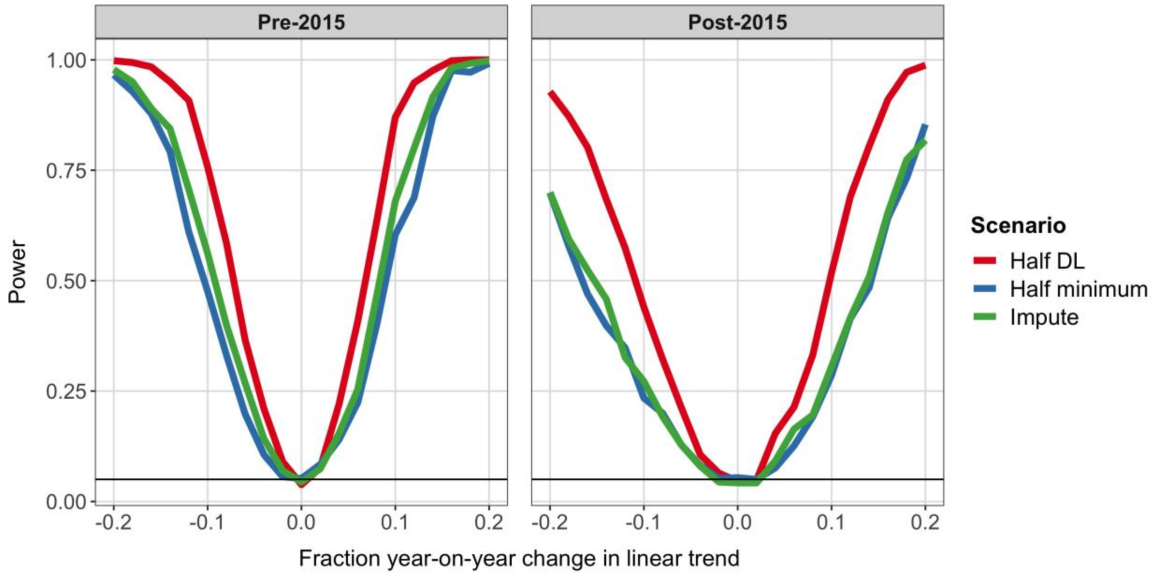

S1 Fig. Bootstrap power results for below detection limit (BDL) imputation investigation for NO_x constituent in the Burdekin study area. Power curves for comparing method of imputing values of below detection limit values for nitrite and nitrate prior to summation to form the NO_x analyte. All data before 2015-01-01 were used in the pre-2015 analysis and for the post-2015 analysis. No sub setting of the pre-2015 data were performed for this analysis as we were interested in comparing the imputation methods rather than comparing power between sampling regimes. Power in these plots refers to the linear trend component and is from a regional analysis of NO_x from the Burdekin study area. Other study area showed similar differences in power curves between BDL imputation methods. The imputation scenarios are: impute the BDL with half the detection limit (Half DL): impute the BDL values with half the minimum value over all measurements (Half minimum); and impute the BDLs using sampled values from a log-normal distribution with mean and variance parameters estimated using the rROS method (Impute). The x-axis corresponds to the simulated fractional year-on-year changes that ranged from $\delta = (-0.2, -0.18, \ldots, 0.18, 0.2)$. Darker grey horizontal line represents 80% power.
