# Supplementary material for: Sampling re-design increases power to detect change in the Great Barrier Reef’s inshore water quality
Source: PLoS One. 2022 Jul 28;17(7):e0271930. doi: 10.1371/journal.pone.0271930 (PMC9333274; doi:10.1371/journal.pone.0271930)
Supplement: S2 Fig — (PDF) [file pone.0271930.s004.pdf]

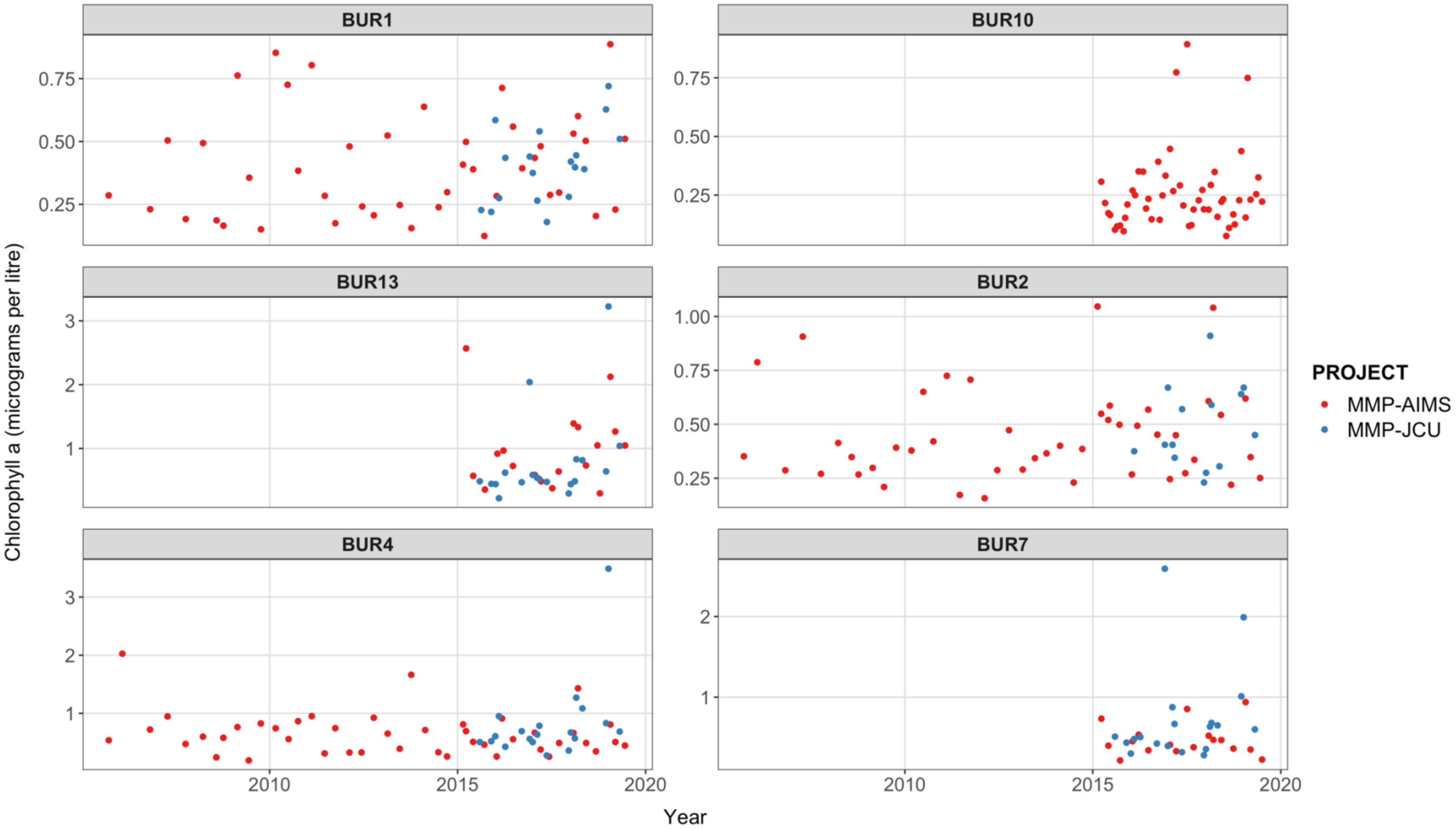

S2 Fig. Time series of Chlorophyll *a* (Chl-*a*) concentrations for the five sampling locations in the Burdekin study area. Grab samples on which Chl-*a* concentrations were measured were collected and analysed by AIMS (red) and JCU (blue). Panel headings correspond to the abbreviated names for each of the sampling locations within the Burdekin study area.
