# Supplementary material for: Sampling re-design increases power to detect change in the Great Barrier Reef’s inshore water quality
Source: PLoS One. 2022 Jul 28;17(7):e0271930. doi: 10.1371/journal.pone.0271930 (PMC9333274; doi:10.1371/journal.pone.0271930)
Supplement: S3 Fig — (PDF) [file pone.0271930.s005.pdf]

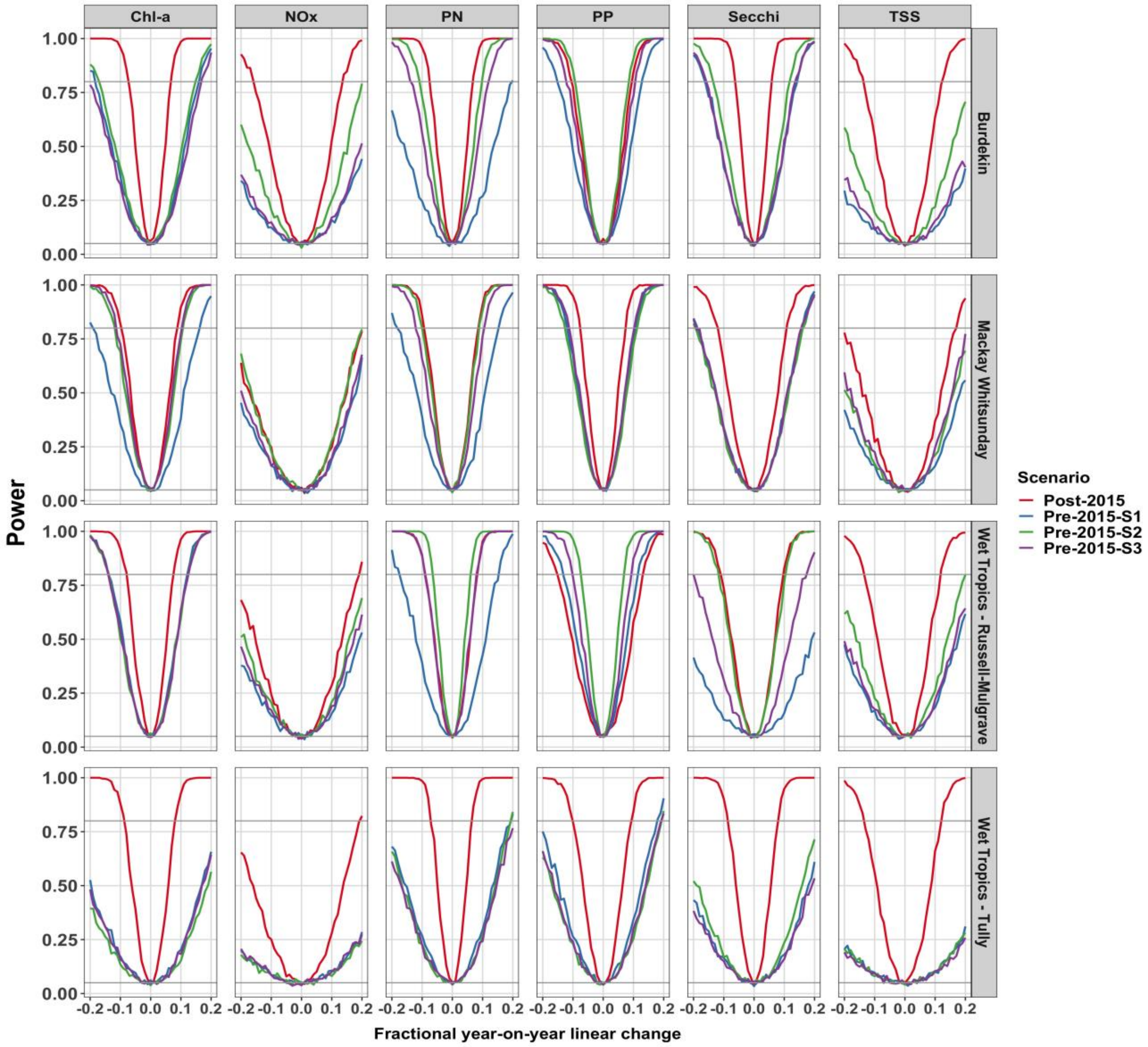

S3 Fig. Power curves from pre- and post-2015 sampling design comparisons for six water quality analytes across four sampling areas. Each line represents the power curve from the bootstrap resampling method for each of the time-series scenarios namely: data after 2015-01-01 (Post-2015); data with dates greater than 2005-09-18 and less than 2009-12-30 (Pre-2015-S1), greater than or equal to 2010-06-15 and less than 2015-01-01 (Pre-2015-S2) ; and between 2008-02-01 and 2012-05-09 (Pre-2015-S3). The x-axis corresponds to the simulated fractional year-on-year changes that ranged from $\delta = (-0.2, -0.19, \ldots, 0.19, 0.2)$. Darker grey horizontal line represents 80% power. Power curves are presented for Chlorophyll *a* (Chl-*a*), nitrogen oxide (NO_x), particulate nitrogen (PN), particulate phosphorus (PP), Secchi depth (Secchi), and total suspended solids (TSS), for the Burdekin, Mackay-Whitsundays, Russell-Mulgrave and Tully study areas.
