# Supplementary material for: Sampling re-design increases power to detect change in the Great Barrier Reef’s inshore water quality
Source: PLoS One. 2022 Jul 28;17(7):e0271930. doi: 10.1371/journal.pone.0271930 (PMC9333274; doi:10.1371/journal.pone.0271930)
Supplement: S4 Fig — (PDF) [file pone.0271930.s006.pdf]

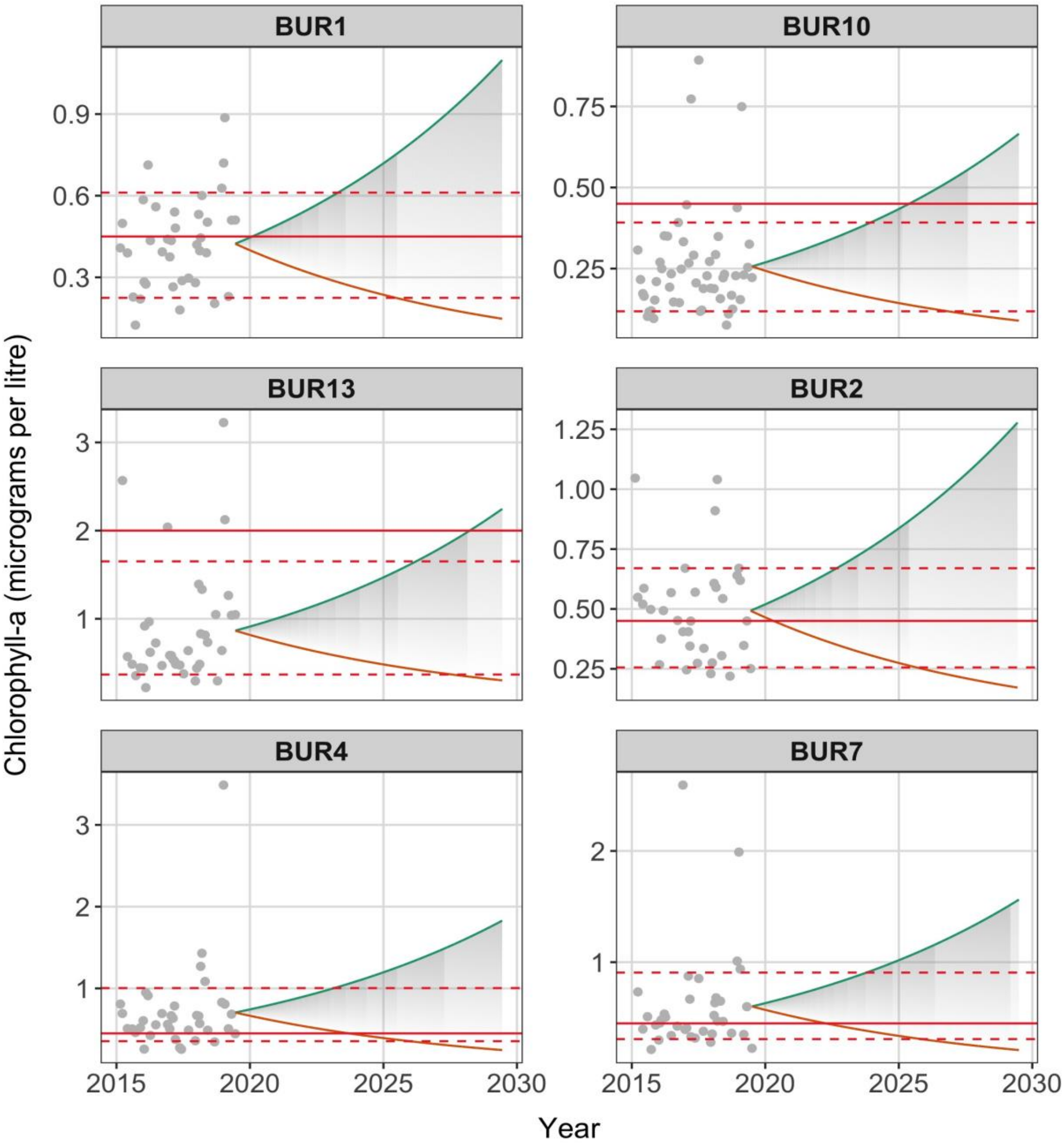

S4 Fig. Time to exceedance of 10th and 90th percentile for Chl-*a* for six sampling locations in the Burdekin study area. In each panel, the red solid line is the water quality guideline value, which is specific to the analyte and study area (taken from Supplementary Table D-5 in (Gruber *et al.*, 2020). Red dotted lines correspond to 10th and 90th percentile of the five years of post-2015 data (grey dots). Grey shaded area indicates area where we expect trends of less than a fractional change of 0.1 to lie. The green (increasing 10% year on year change) and orange (decreasing 10% year on year change) lines are computed as median(y)×$(1 - \delta)^{x/T}$ or mean(y)×$(1 - \delta)^{x/T}$ depending on whether the guideline value is compared against the mean or median and y corresponds to the vector of post-2015 data within sampling location, $x = (1/T, 2/T \dots, 10T)$ and $T = 365.5$. Panel headings correspond to the abbreviated names of each of the sampling location within the Burdekin study area.
