# Supplementary material for: Sampling re-design increases power to detect change in the Great Barrier Reef’s inshore water quality
Source: PLoS One. 2022 Jul 28;17(7):e0271930. doi: 10.1371/journal.pone.0271930 (PMC9333274; doi:10.1371/journal.pone.0271930)
Supplement: S5 Fig — (PDF) [file pone.0271930.s007.pdf]

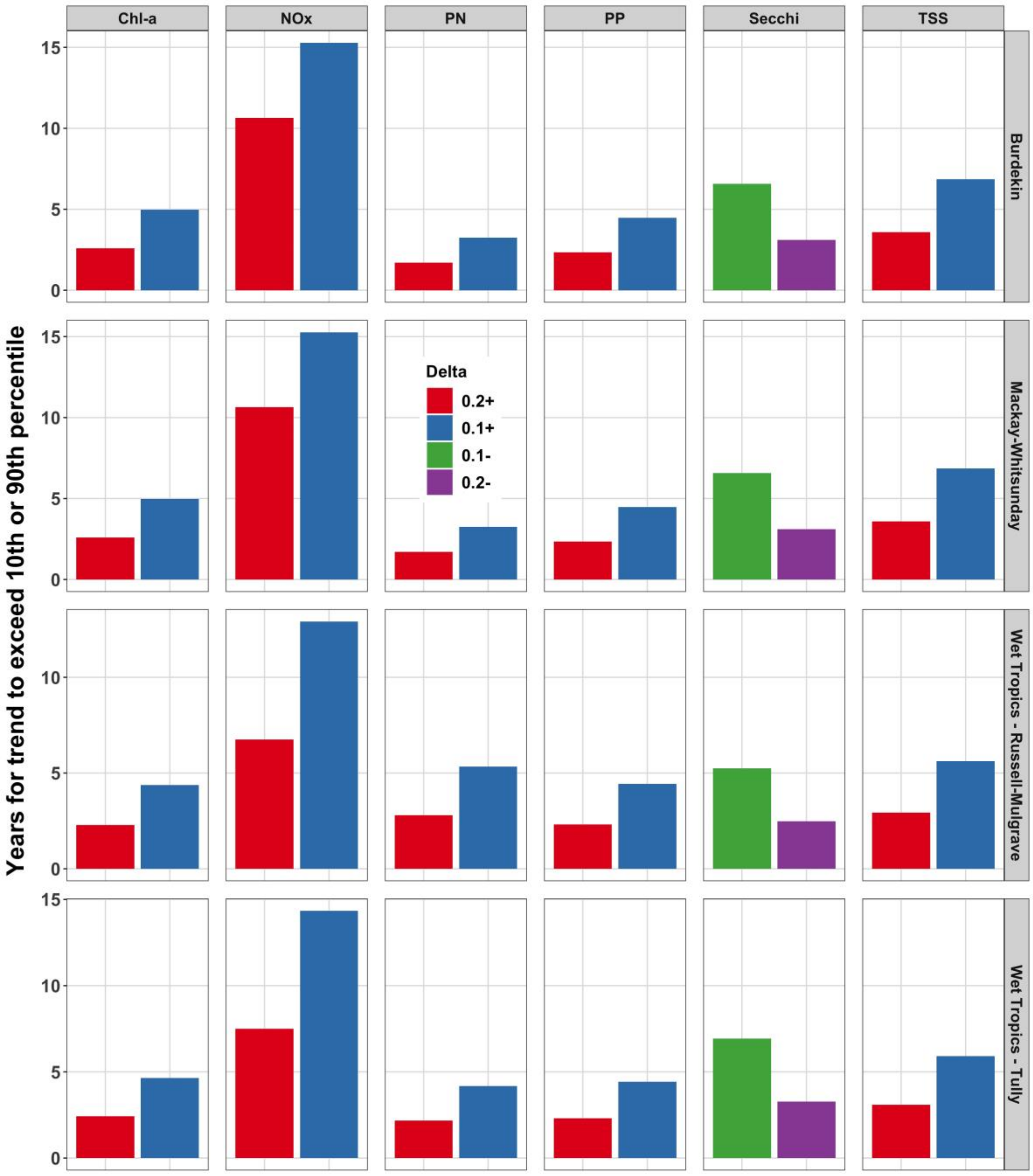

S5 Fig. Time (in years) for linear trend to exceed the 10th and 90th percentile for six water quality analytes across four sampling areas at a 0.20 or 0.10 fractional year-on-year change. In each panel, the height of each bar represents the time for the trend component at a specific fractional change (e.g., 0.1+:- increasing at 0.1 fractional change or 0.2- :- decreasing at a 0.2 fractional change) to exceed the 90th percentile or 10th percentile (for Secchi depth, with decreasing values implying worse water quality) the five years of post-2015 data (see Supplementary Figure 4 for example calculation). The columns are presented for Chlorophyll *a* (Chl-*a*), nitrate/nitrite (NO_x), particulate nitrogen (PN), particulate phosphorus (PP), Secchi depth (Secchi), and total suspended solids (TSS), for the Burdekin, Mackay-Whitsundays, Russell-Mulgrave and Tully study areas.
