# Supplementary material for: Sampling re-design increases power to detect change in the Great Barrier Reef’s inshore water quality
Source: PLoS One. 2022 Jul 28;17(7):e0271930. doi: 10.1371/journal.pone.0271930 (PMC9333274; doi:10.1371/journal.pone.0271930)
Supplement: S8 Fig — (PDF) [file pone.0271930.s010.pdf]

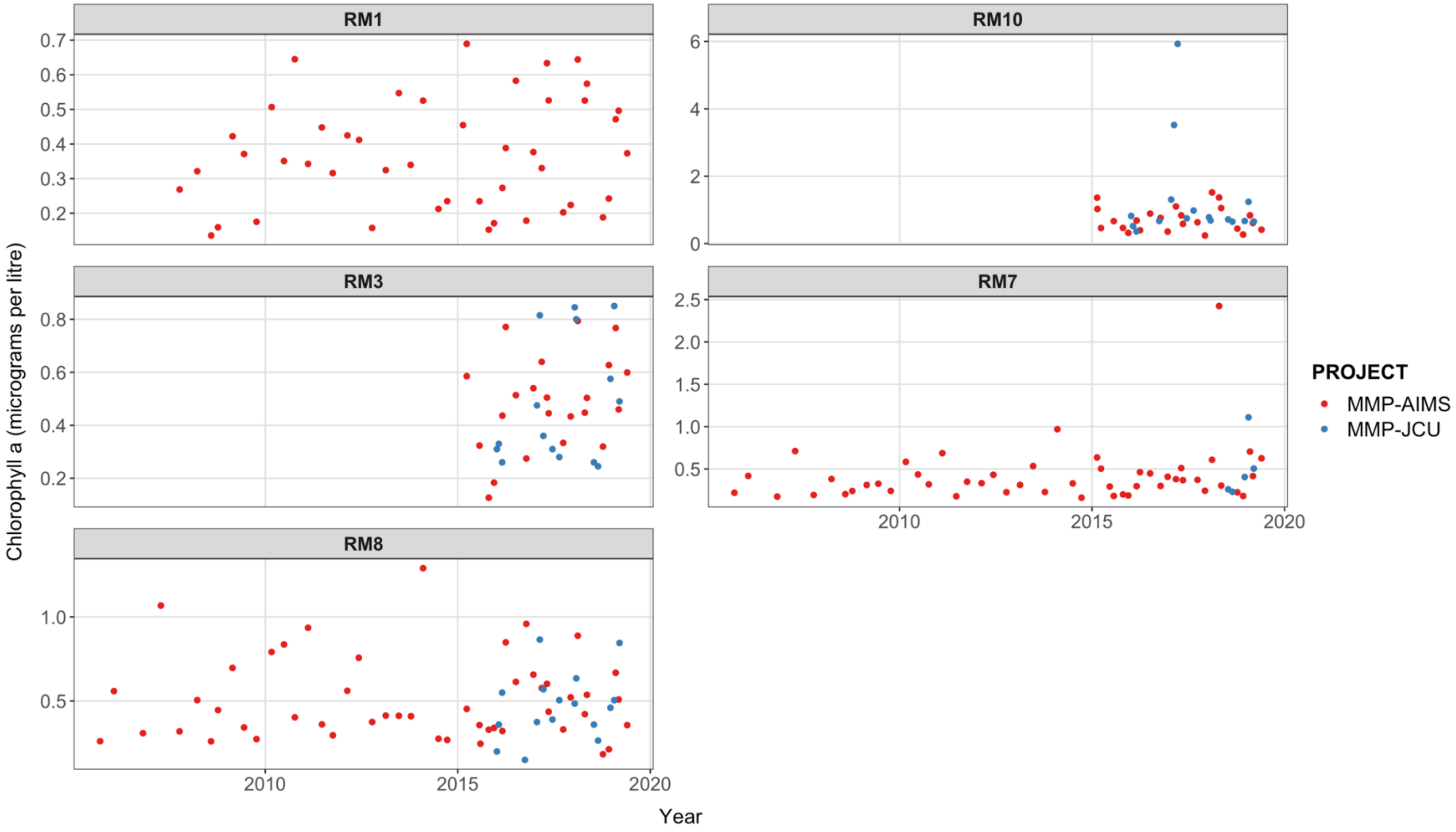

S8 Fig. Time series of Chlorophyll *a* (Chl-*a*) concentrations for the five sampling locations in the Russell-Mulgrave study area, within the Wet Tropics Natural Resource Management region. Grab samples on which Chl-*a* concentrations were measured were collected and analysed by AIMS (red) and JCU (blue). Panel headings correspond to the abbreviated names for each of the sampling location within the Russell-Mulgrave study area.
