# Supplementary material for: Sampling re-design increases power to detect change in the Great Barrier Reef’s inshore water quality
Source: PLoS One. 2022 Jul 28;17(7):e0271930. doi: 10.1371/journal.pone.0271930 (PMC9333274; doi:10.1371/journal.pone.0271930)
Supplement: S9 Fig — (PDF) [file pone.0271930.s011.pdf]

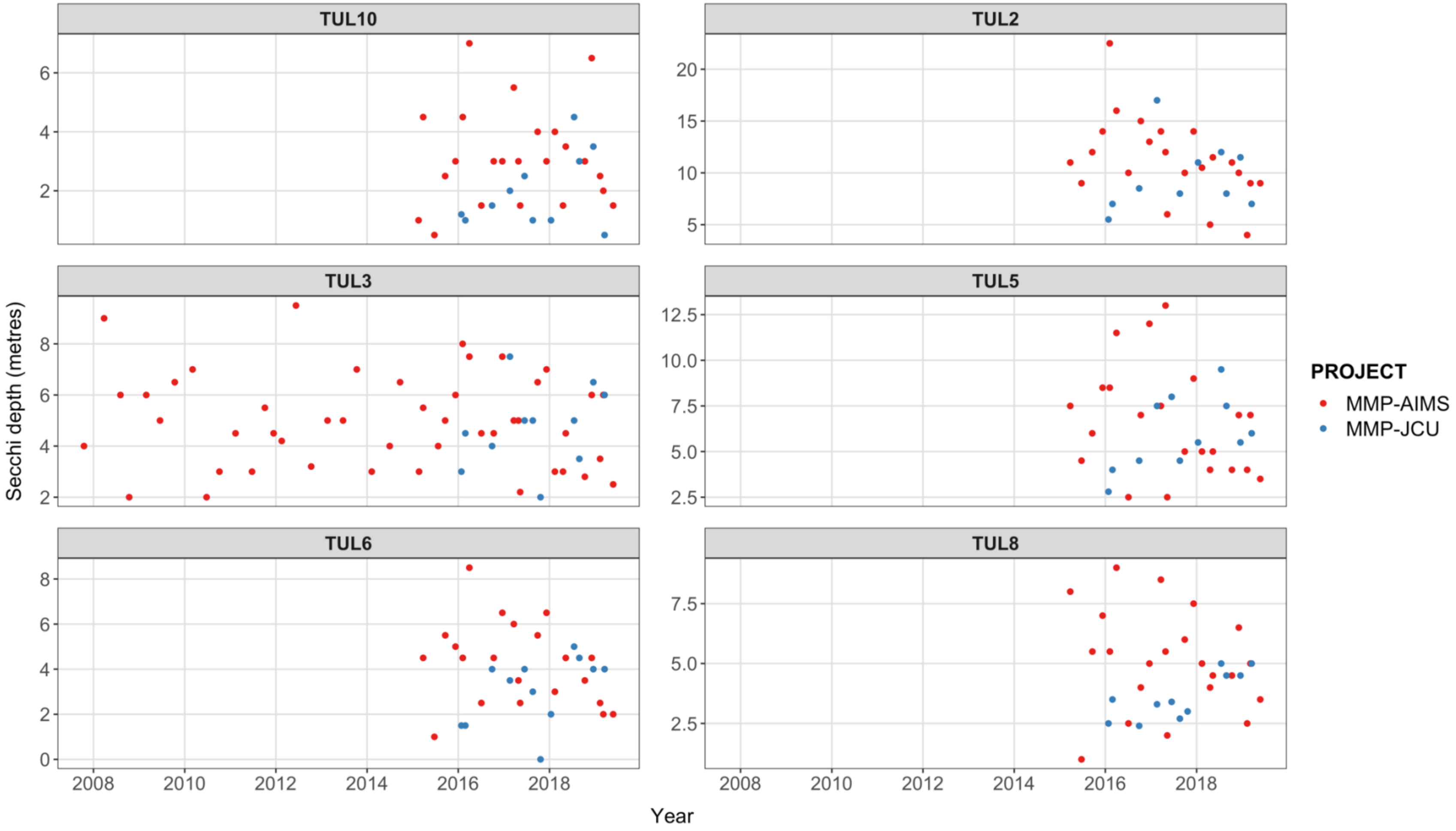

S9 Fig. Time series of Secchi depth for the six sampling locations in the Tully study area, within the Wet Tropics Natural Resource Management region. Secchi depth measurements were conducted by AIMS (red) and JCU (blue). Panel headings correspond to the abbreviated names for each of the sampling location within the Tully study area.
