# Supplementary material for: Sampling re-design increases power to detect change in the Great Barrier Reef’s inshore water quality
Source: PLoS One. 2022 Jul 28;17(7):e0271930. doi: 10.1371/journal.pone.0271930 (PMC9333274; doi:10.1371/journal.pone.0271930)
Supplement: S12 Fig — (PDF) [file pone.0271930.s014.pdf]

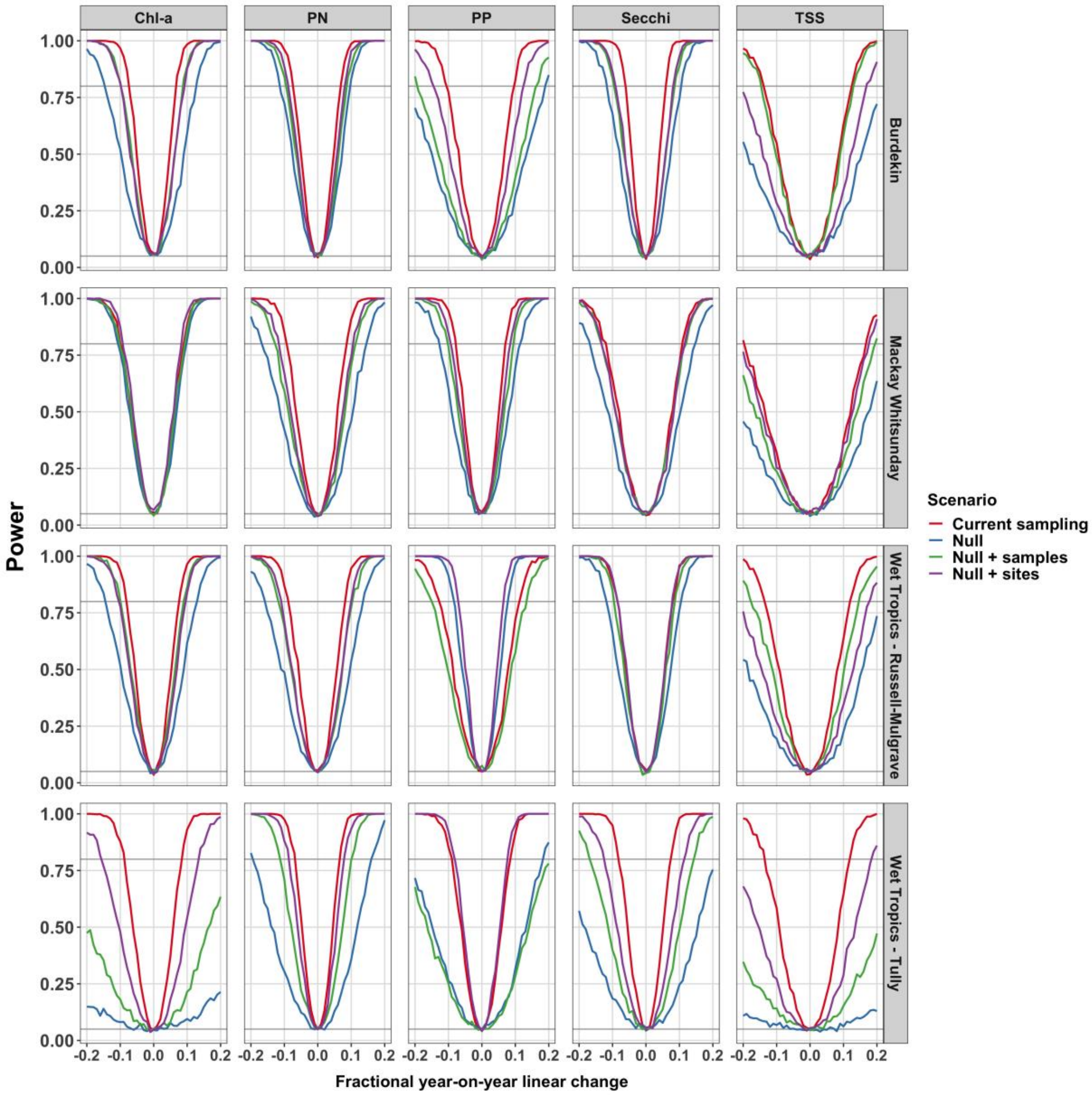

S12 Fig. Power curves for investigating the change in power due to increasing sampling frequency or adding sampling locations. Each line represents the power curve from the bootstrap resampling method for each of the subsampled data scenarios namely: after 201501-01 data (current sampling) into the following sets: 1) we down sampled the post-2015 data to those sites used in the pre-2015 sampling regime and a similar within year sampling density to pre-2015, which is three time per year (null); 2) retain the same within year sampling density but add in the extra sites for each study area to the post-2015 data (null + sites); and 3) retain the pre-2015 sites but increase the sampling density to the post-2015 data (null + sites). The x-axis corresponds to the simulated fractional year-on-year changes that ranged from $\delta = (-0.2, -0.19, \ldots, 0.19, 0.2)$. Darker grey horizontal line represents 80% power. Power curves are presented for Chlorophyll *a* (Chl-*a*), nitrate/nitrite (NO_x), particulate nitrogen (PN), particulate phosphorus (PP), Secchi depth (Secchi), and total suspended solids (TSS), for the Burdekin, Mackay-Whitsundays, Russell-Mulgrave and Tully study areas.
